# siRNA biogenesis and advances in topically applied dsRNA for controlling virus infections in tomato plants

**DOI:** 10.1038/s41598-020-79360-5

**Published:** 2020-12-17

**Authors:** Camila M. Rego-Machado, Erich Y. T. Nakasu, João M. F. Silva, Natália Lucinda, Tatsuya Nagata, Alice K. Inoue-Nagata

**Affiliations:** 1 Department of Plant Pathology, University of Brasília, Federal District, Brazil; 2 Laboratory of Virology and Molecular Biology, Embrapa Vegetables, Federal District, Brazil; 3 Department of Molecular Biology, University of Brasília, Federal District, Brazil; 4 Present Address: Department of Plant Pathology, University of Florida, Florida, USA

**Keywords:** Biological techniques, Biotechnology, Plant sciences

## Abstract

A non-transgenic approach based on RNA interference was employed to induce protection against tomato mosaic virus (ToMV) infection in tomato plants. dsRNA molecules targeting the *cp* gene of ToMV were topically applied on plants prior to virus inoculation. Protection was dose-dependent and sequence-specific. While no protection was achieved when 0–16 µg dsRNA were used, maximum rates of resistance (60 and 63%) were observed in doses of 200 and 400 µg/plant, respectively. Similar rates were also obtained against potato virus Y when targeting its *cp* gene. The protection was quickly activated upon dsRNA application and lasted for up to 4 days. In contrast, no detectable antiviral response was triggered by the dsRNA from a begomovirus genome, suggesting the method is not effective against phloem-limited DNA viruses. Deep sequencing was performed to analyze the biogenesis of siRNA populations. Although long-dsRNA remained in the treated leaves for at least 10 days, its systemic movement was not observed. Conversely, dsRNA-derived siRNA populations (mainly 21- and 22-nt) were detected in non-treated leaves, which indicates endogenous processing and transport through the plant. Altogether, this study provides critical information for the development of novel tools against plant viruses; strengths and limitations inherent to the systems are discussed.

## Introduction

Tomato (*Solanum lycopersicum*) is one of the main vegetables grown in the world, but the occurrence of several plant diseases, particularly those of viral etiology, frequently causes substantial production losses in this crop. Recently, the development of virus-resistant plants has been successfully achieved via transgenic approaches by expression of double-stranded RNA (dsRNA) molecules homologous to genomic regions of viruses, i.e., through activation of the RNA interference (RNAi) mechanism^1,2^. Once present in the plant cells, long dsRNA molecules are cleaved by Dicer-like (DCL, RNAse III family) enzymes into 21–24-nt small interfering RNA (siRNA) or micro RNA (miRNA) duplexes, depending on the origin of the molecule and the downstream pathways involved^3,4^. Both miRNA and siRNA are sorted by Argonaute (AGO) proteins, mostly based on the 5′-nucleotide identity; and one of the two small RNA strands is loaded and incorporated into the RNA-induced silencing complex (RISC)^5,6^. Then, AGO proteins either mediate post-transcriptional gene silencing (PTGS) via the cleavage of complementary transcripts, or mediate transcriptional gene silencing (TGS) via DNA methylation^7,8^. Thus, RNAi plays an important role in plant defense against pathogens and is a powerful tool to control viruses in cultivated plants^9^.

RNAi-mediated viral resistance also requires host RNA-dependent RNA polymerases (RDR) to produce secondary viral siRNA molecules, thus amplifying the antiviral response^10,11^. Subsequent cell-to-cell dispersal and phloem translocation of siRNA are also important aspects for RNAi-based protection of the whole plant^12^.

New techniques for dsRNA large-scale production have recently emerged^13–15^, and the topical application of these molecules for plant protection against viruses has been attempted as an alternative to transgenic plants^16,17^ in order to avoid the cumbersome steps of plant transformation, screening, and biosafety issues. Indeed, a number of studies have shown that exogenous dsRNA targeting viral sequences produced either in vitro or in vivo conferred protection against the cognate plant viruses, including members of *Tobamovirus, Potyvirus*, *Alfamovirus,* and *Potexvirus*^18–22^.

Based on the premise that exogenous dsRNA molecules applied on the surface of plants activate endogenous RNA defense machinery against homologous viruses, in a way of a vaccination effect, our final aim is to develop a method to control virus diseases by dsRNA topical application. Here, we used an RNAi-mediated non-transgenic approach to test resistance induction in tomatoes against two single-stranded RNA viruses, tomato mosaic virus (ToMV, genus *Tobamovirus*) and potato virus Y (PVY, *Potyvirus*), and a single-stranded DNA virus, tomato severe rugose virus (ToSRV, *Begomovirus*). ToMV was used as the model system for testing the effectiveness of the methodological strategy. We evaluated dose, specificity and durability of protection, application method, dsRNA systemic transport, and siRNA analyzes by deep sequencing. More specifically, we confirmed that the dsRNA-mediated antiviral resistance is sequence-specific and dose-dependent. The strategy significantly reduced infection rates by ToMV and PVY, but not ToSRV. We also demonstrate dsRNA processing and movement of siRNA molecules within plants following the dsRNA application method that leads to improved protection of tomato plants.

## Results

### Inhibition of ToMV-related local lesions development in hypersensitive hosts

**Figure 1:**
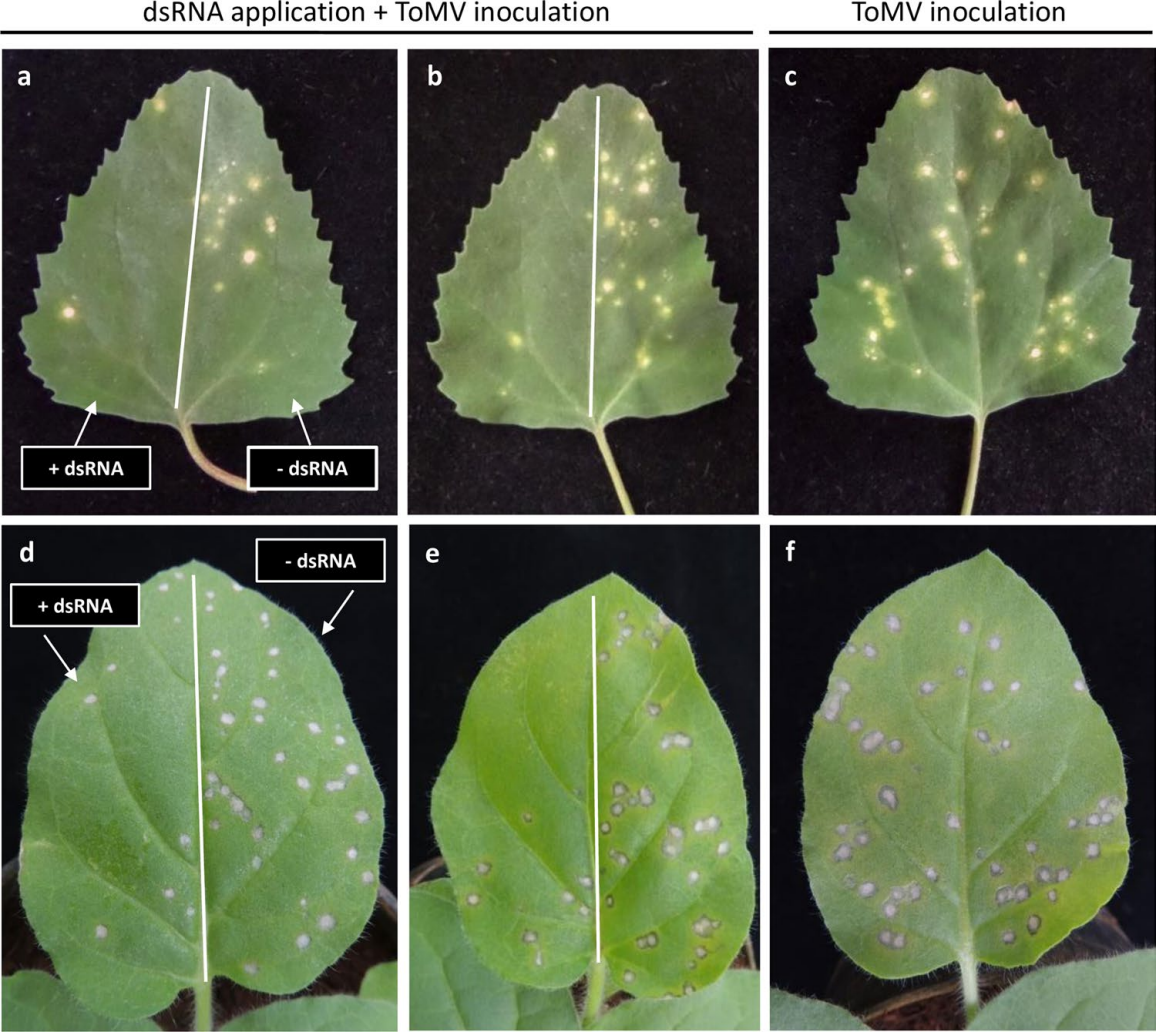
*Chenopodium quinoa* (**a**–**c**) and *Nicotiana glutinosa* (**d**–**f**) leaves with local lesions 4 days after mechanical inoculation of tomato mosaic virus (ToMV). dsRNA of ToMV-*cp* gene (**a**,**d**) and ToMV-*mp* gene (**b**,**e**) were mechanically applied on the left halves simultaneously with ToMV; the right halves were treated with DEPC-water and ToMV. As a control, only ToMV was inoculated on both halves of the leaves in (**c**,**f**).

Preliminary trials for testing the dsRNA ability to induce protection against a viral infection were carried out on *Chenopodium quinoa* and *Nicotiana glutinosa* plants using in vitro produced dsRNA molecules homologous to the *cp* and *mp* genes of ToMV. Fully developed leaves were selected and one half was inoculated with ToMV while the opposite half was inoculated with the virus and dsRNA. Figure 1 shows a typical reaction at 4 days post-inoculation (dpi). When ToMV inoculum was applied in either plant, chlorotic/necrotic local lesions appeared scattered in the entire leaf blade (Fig. 1c,f). By simultaneous application of dsRNA, the number of lesions was clearly reduced (Fig. 1a,b,d,e; Supplementary Fig. S1), implying that the presence of dsRNA at least partially blocked the infection process. Both dsRNAs of the *cp* and *mp* genes promoted a similar reduction of local lesions (Fig. 1; Supplementary Fig. S1). The dsRNA of the *cp* gene was selected for the further experiments with ToMV in tomato plants as it induced a slightly lower average number of lesions in both hypersensitive hosts (Supplementary Fig. S1), and because it is commonly used in RNAi-based resistance reports^14,15,21,23–26^.

### Protection against ToMV infection by dsRNA topical application

**Figure 2:**
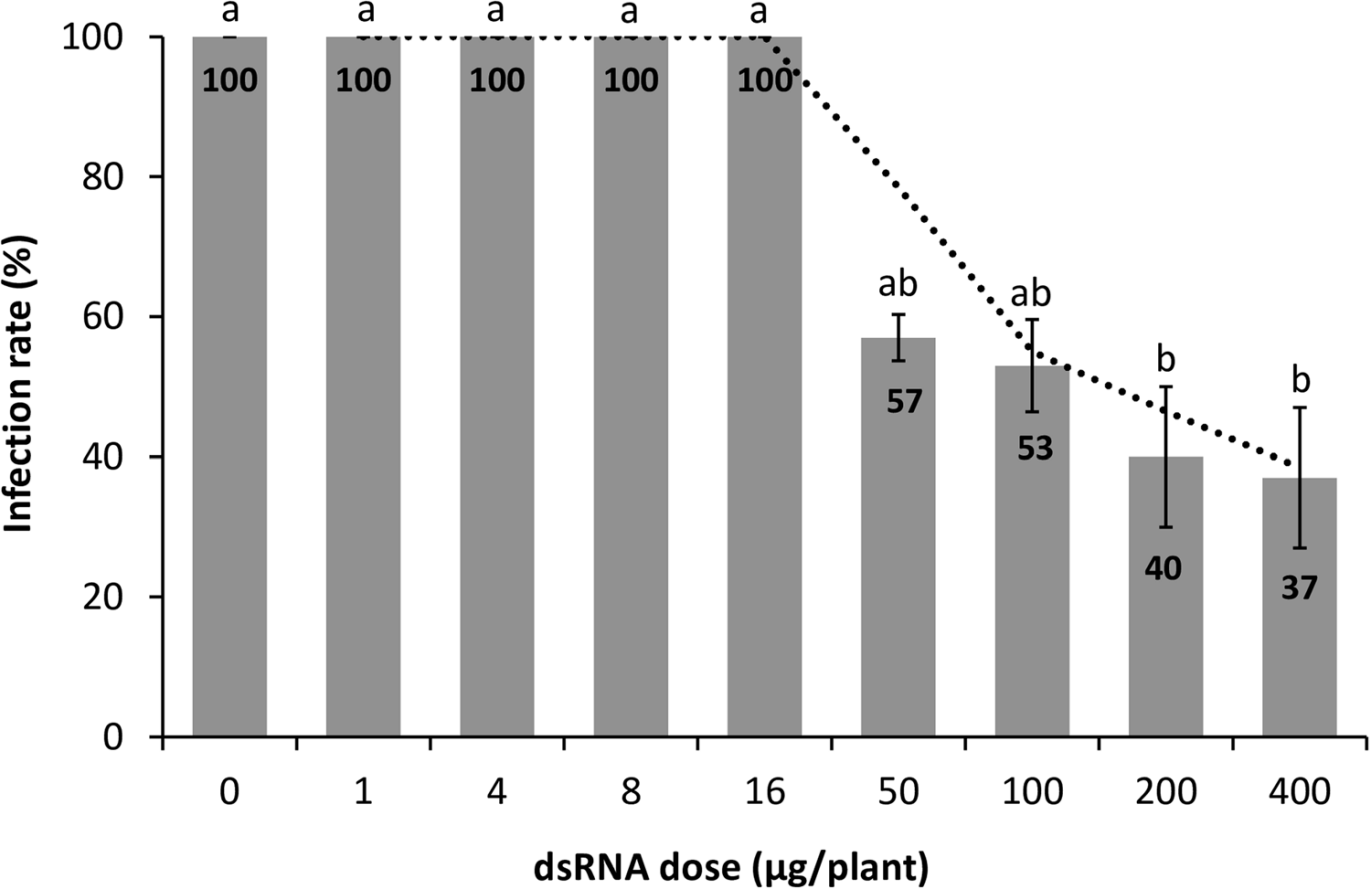
Effects of the application of dsRNA molecules homologous to tomato mosaic virus (ToMV) sequence on the viral infection rate in tomato plants. Testing the doses of dsRNA at 0, 1, 4, 8, 16, 50, 100, 200, and 400 µg per plant. ToMV was mechanically inoculated 24 h after dsRNA application. Infection rate was determined at 7 dpi using the number of positive plants detected by indirect-ELISA, and calculated based on the number of infected plants out of total plants used in each treatment. The results are expressed as average values of 10 to 12 plants in 3 independent trials. Bars represent the respective standard errors. Letters above error bars indicate significantly different results based on Tukey test (*p* < 0.05). The dotted line is an infection rate trend line.

The ability of dsRNA molecules to induce protection to ToMV was tested in tomato plants using a commercially synthetized dsRNA. Initially, a dose–response curve was determined according to the dsRNA amount. ToMV-dsRNA (0, 1, 4, 8, 16, 50, 100, 200, and 400 µg per plant) was mechanically applied on the plants, and ToMV was inoculated 24 h later. Before the expression of clear symptoms, the serological test performed at 7 dpi showed that 100% of the plants were systemically infected when 0 (positive control) to 16 µg of dsRNA were used (Fig. 2). From 50 µg upwards the infection rate was progressively reduced with increasing dsRNA amounts, ranging from 57 to 37% of infected plants (Fig. 2). The highest protection was achieved when 200 and 400 µg were applied, with infection rates of 40 and 37%, respectively (Fig. 2). Based on these results, 200 µg/plant was defined as the standard dose for all further tests.

The development of symptoms was monitored daily up to 20 dpi. No difference was observed concerning the time interval for the appearance of initial symptoms in the infected plants of all treatments (ELISA-positive plants at 7 dpi). The first symptomatic plants showed mild chlorosis and leaf deformation at 8 dpi; over time the symptoms evolved to mosaic. However, positive controls expressed stronger symptoms of clear mosaic, stunting, and leaf narrowing associated with pointed tips at 15 dpi. Interestingly, the dsRNA-treated plants that became infected showed milder symptoms than dsRNA-untreated plants (positive control; Supplementary Fig. S2), suggesting that some level of dsRNA protection occurred. ELISA-negative plants (dsRNA-treated or dsRNA-untreated) showed no symptoms and were not infected, even after 20 dpi.

### Evaluation of dsRNA application procedure on plants

**Figure 3:**
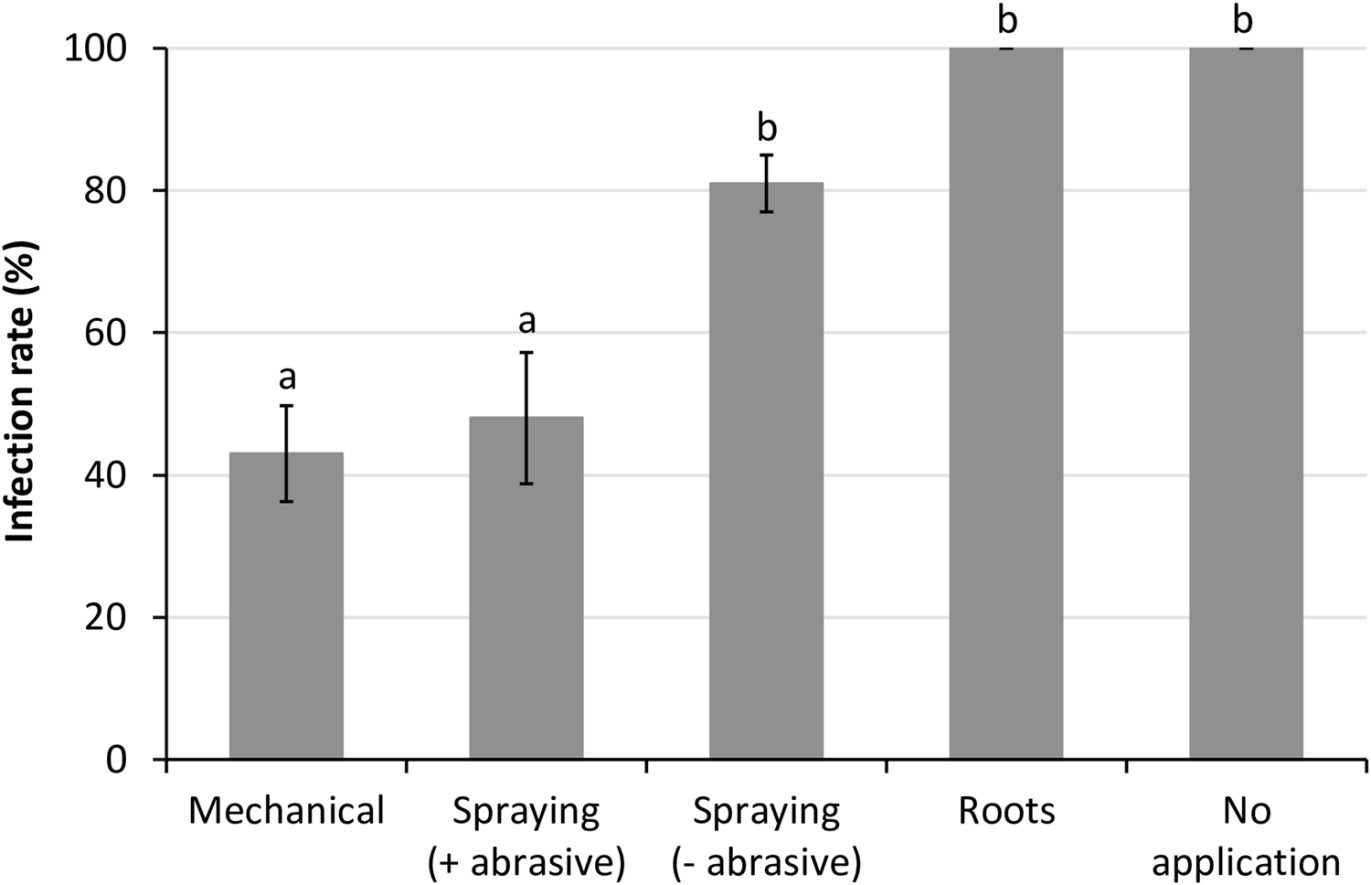
Effects of the application of dsRNA molecules homologous to tomato mosaic virus (ToMV) sequence on the viral infection rate in tomato plants. Testing application methods of ToMV-dsRNA: mechanical, spraying with abrasive (+ abrasive), spraying without abrasive (– abrasive), and root immersion in dsRNA solution (roots). The positive control consisted of non-treated plants (no dsRNA application). ToMV was mechanically inoculated 24 h after application. Infection rate was determined at 7 dpi using the number of positive plants by indirect-ELISA, and calculated based on the number of infected plants out of total plants used in each treatment. The results are expressed as average values of 10 to 12 plants in 3 independent trials. Bars represent the respective standard errors. Letters above error bars indicate significantly different results based on Tukey test (*p* < 0.05).

Different application procedures were tested to determine interference with the protection induction, concerning the final aim of field use. Four application methods were analyzed using ToMV-dsRNA: mechanically, spraying with and without abrasive, and root immersion in a dsRNA-containing solution. Mechanical application and spraying with an abrasive were the most efficient methods, with the mean value of three independent trials of 57 and 52% protection, respectively (Fig. 3). Spraying the dsRNA without the abrasive induced only 19% resistance and did not significantly differ (*p* > 0.05) from the control treatment (Fig. 3). Similarly, the root immersion strategy was not efficient in protecting the plants against infection (Fig. 3). Since the spraying plus abrasive and the mechanical methods did not differ, we defined the mechanical application as the standard procedure.

### Specificity of the protection induced by dsRNA

**Figure 4:**
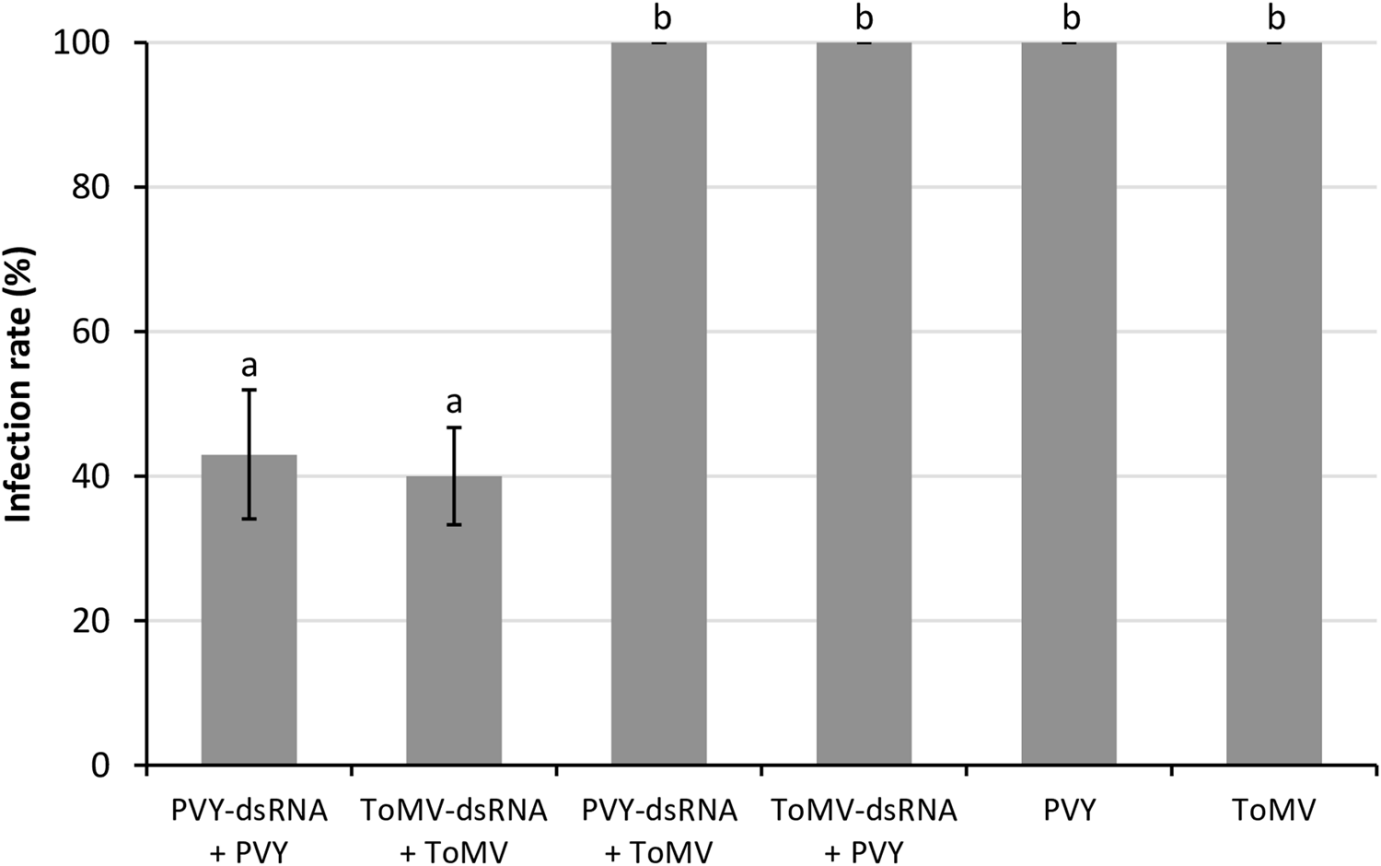
Effects of the application of dsRNA molecules homologous to tomato mosaic virus (ToMV) and potato virus Y (PVY) sequences on the viral infection rate in tomato plants. Testing specificity of protection by PVY-dsRNA and ToMV-dsRNA application. The dsRNA was mechanically applied and then PVY or ToMV was inoculated. Infection rate was determined at 7- and 10-dpi for ToMV and PVY, respectively, using the number of positive plants by indirect-ELISA, and calculated based on the number of infected plants out of total plants used in each treatment. The results are expressed as average values of 10 to 12 plants in 3 independent trials. Bars represent the respective standard errors. Letters above the error bars indicate significantly different results based on Tukey test (*p* < 0.05).

In order to evaluate the specificity of protection, dsRNA was synthesized based on the CP-sequence of PVY and tested for protection induction in tomato plants. Plants treated with PVY-dsRNA were mechanically inoculated with PVY. A detection test at 10 dpi revealed that the dsRNA induced resistance to PVY in 57% of the plants (Fig. 4); this rate was similar to the combination ToMV-dsRNA against ToMV infection (Fig. 4). When plants were inoculated with ToMV post PVY-dsRNA treatment, no protection was observed and 100% of the plants became infected (Fig. 4), indicating that resistance is specific to the applied dsRNA. The protection specificity was also observed with the application of ToMV-dsRNA followed by PVY inoculation (Fig. 4).

### Durability of protection after exogenous dsRNA application

**Figure 5:**
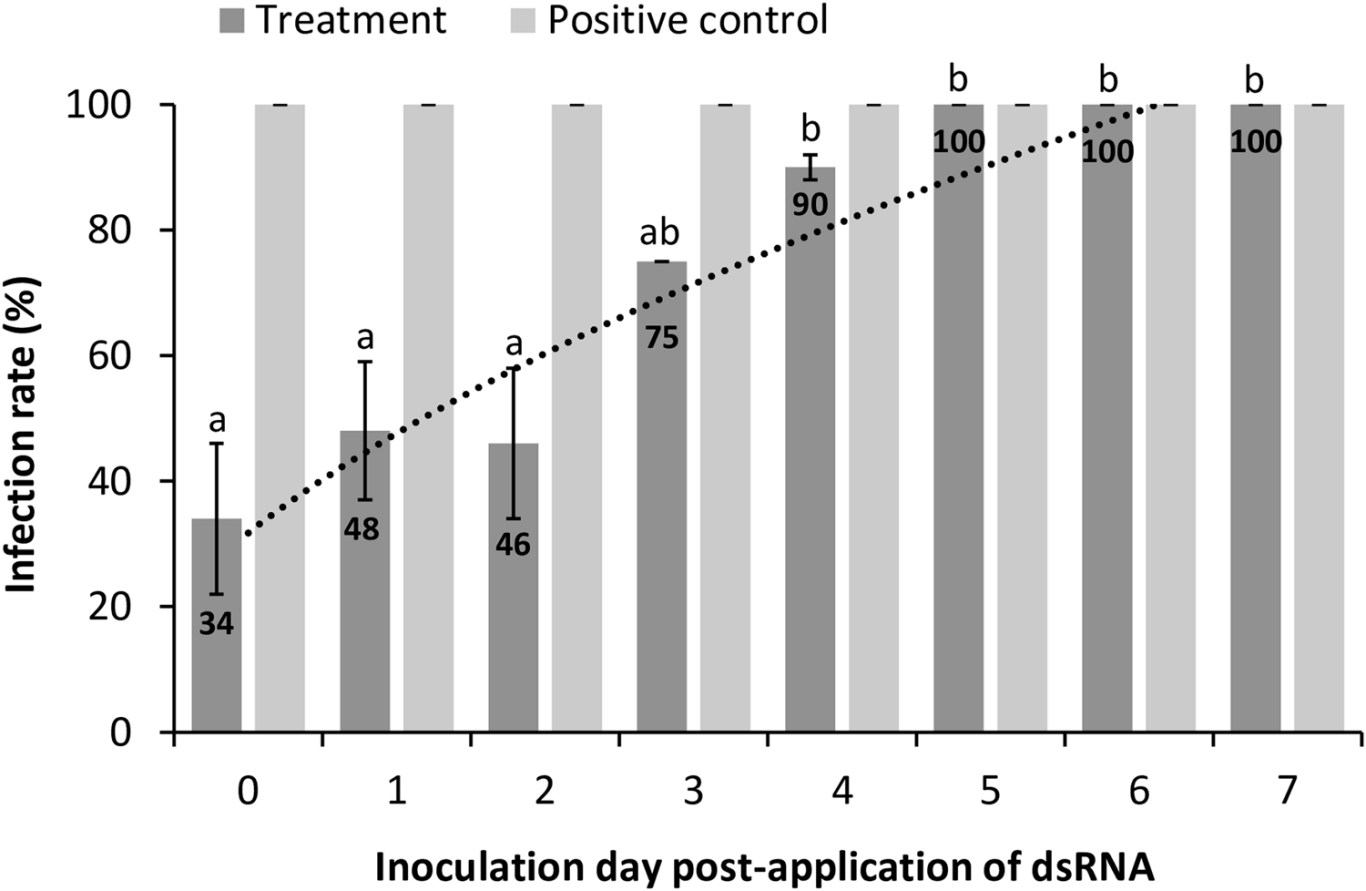
Effects of the application of dsRNA molecules homologous to tomato mosaic virus (ToMV) sequence on the viral infection rate in tomato plants. Durability of the protection after the dsRNA application. Following application on day 0 (zero), ToMV was inoculated daily from 0 (co-application) to 7 days post-treatment. Infection rate was determined at 7 dpi using the number of positive plants by indirect-ELISA, and calculated based on the number of infected plants out of total plants used in each treatment. The results are expressed as average values of 10 to 12 plants in 2 independent trials. Bars represent the respective standard errors. Letters at the error bars indicate significantly different results based on Tukey test (*p* < 0.05). The dotted line is an infection rate trend line only in dsRNA applied samples. Positive control bars represent a control for verification of the inoculum quality; 100% infection rate means that there was no escape from inoculation in the positive control plants.

Once establishing reproducible protocols for inducing protection against viral infection in tomato plants using dsRNA, we tested the durability of this protection. Following ToMV-dsRNA application on day 0 (zero), ToMV was inoculated daily from 0 (co-application) to 7 days post-treatment (dpt). Different sets of test plants and inocula were used for each time point, including control plants to check the inoculum quality. Plants were sampled at 7 dpi for ELISA testing. When plants were treated with ToMV simultaneously to dsRNA, the infection rate was the lowest with 34% (Fig. 5). The infection rate gradually increased with longer periods after dsRNA application; by ToMV inoculation at day 5, all plants were systemically infected (Fig. 5). Since minimal differences were observed in the resistance among days 0, 1, and 2 (Fig. 5), we concluded that plants exhibited maximum protection for up to 2 dpt and weaker protection on 3 and 4 dpt.

### Systemic transport of long-dsRNA molecules

**Figure 6:**
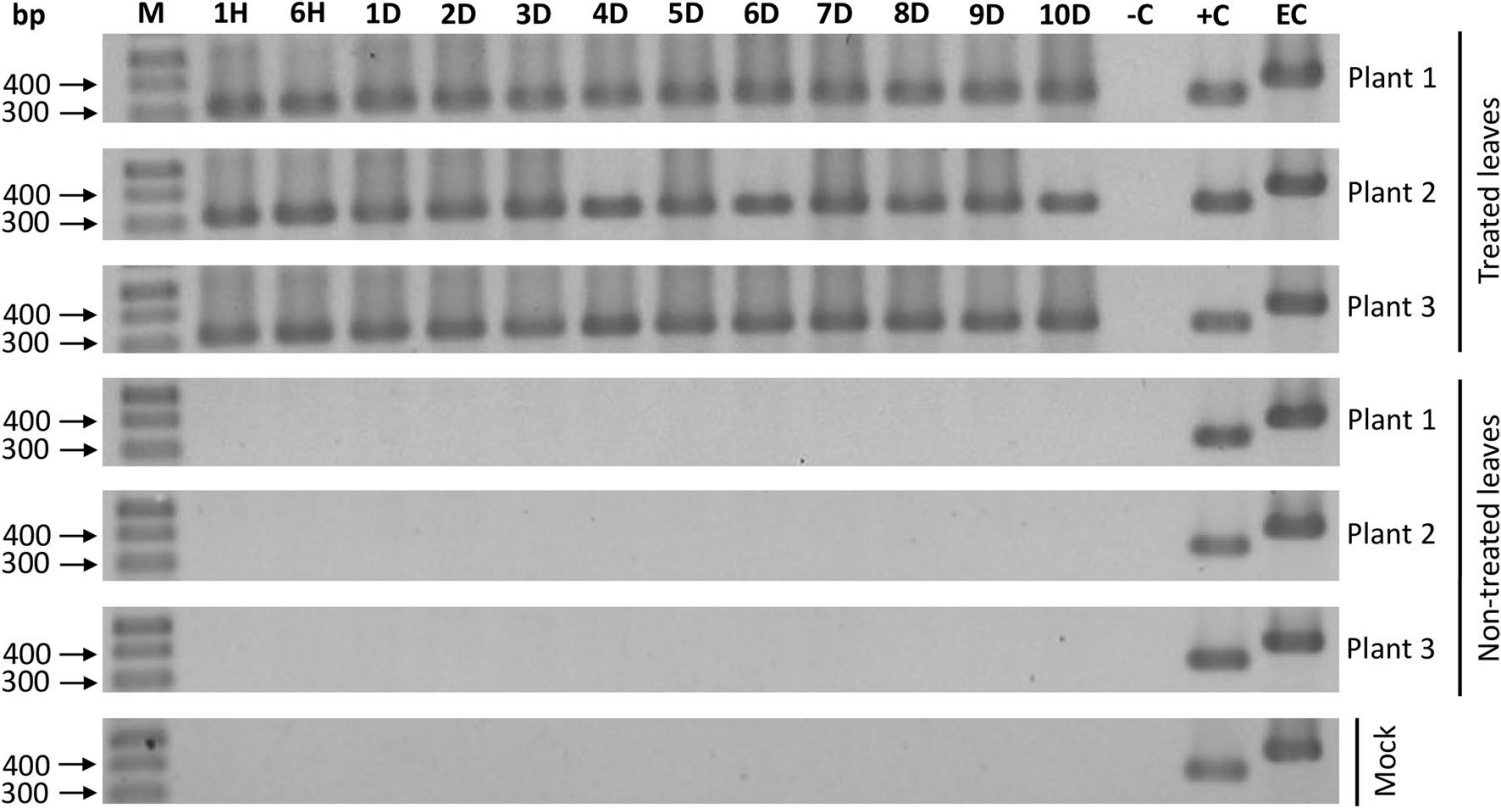
RT-PCR detection of long-dsRNA homologous to tomato mosaic virus (ToMV) sequence in treated and non-treated (younger leaf above the dsRNA application site) leaves of tomato plants at different time points after dsRNA application [1 and 6 h (H), daily from 1 to 10 days (D)] is shown on 1.2% agarose gel electrophoresis. M: molecular weight DNA ladder (1 Kb plus DNA ladder, Thermo Fisher Scientific). –C: RT-PCR negative control (DEPC-water). + C: RT-PCR positive control (ToMV-dsRNA). EC: endogenous control (spliced chloroplast transcript). Full-length gels are presented in Supplementary Fig. S3.

In order to verify the existence of systemic transport and stability of ToMV-dsRNA molecules *in planta*, its presence in treated- and non-treated leaves was monitored over a time course of 1 and 6 h post-treatment (hpt), and daily from 1 to 10 dpt by RT-PCR in triplicate. In the treated leaves, amplicons were detected in all plants and time points, demonstrating that the dsRNA was present and preserved in the leaf blade for at least 10 days (Fig. 6). The dsRNA was not detected in any non-treated leaves at any time point analyzed (Fig. 6). This implies that the long-dsRNA molecules (456 bp) used in this study persisted on the applied leaves, but there was no evidence of systemic movement to the younger parts of the plants.

### Analysis of the siRNA molecules from ToMV genome

The siRNA sequencing by high throughput sequencing (HTS) was performed to evaluate the siRNA populations in the plants generated from the exogenous dsRNA and the virus. The following treatments were analyzed: (i) dsRNA + ToMV(+) [ToMV-dsRNA application + ToMV inoculation, resulting in ToMV-infection], (ii) dsRNA + ToMV(−) [ToMV-dsRNA + ToMV, no infection], (iii) ToMV [DEPC-treated water + ToMV, ToMV-infection], (iv) dsRNA [ToMV-dsRNA + inoculation buffer, no infection], and (v) mock [non-treated plants, no infection]. Illumina sequencing of 15 cDNA libraries yielded 12 to 18 million reads for each. Sequences with sizes ranging from 20- to 25-nt were selected for further bioinformatics analysis from the average values of the three repetitions (each composed of four plants) used in each treatment.

**Figure 7:**
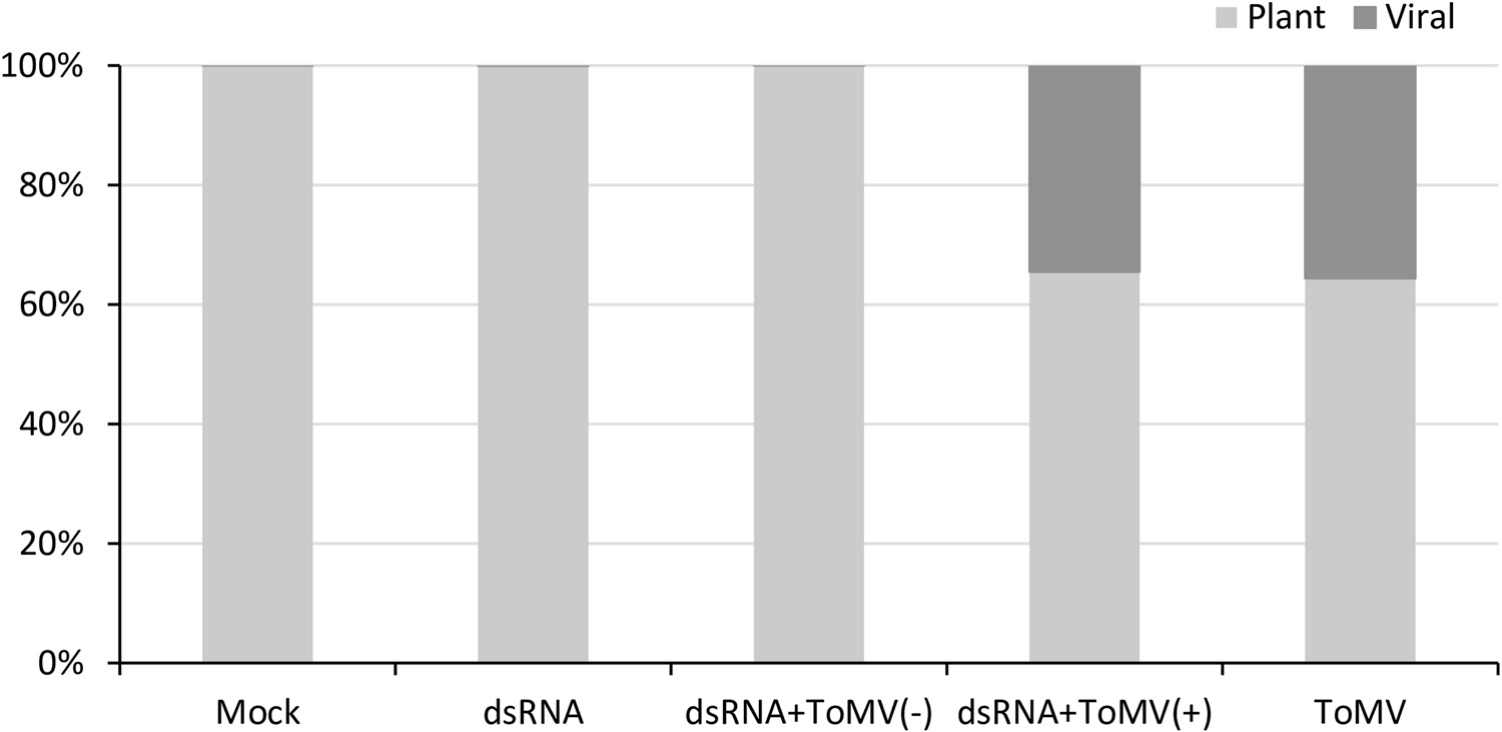
Rate of endogenous and viral small interfering RNA (siRNA) molecules in tomato plants. The siRNA libraries from five treatments [mock, dsRNA, dsRNA + ToMV(−), dsRNA + ToMV(+), and ToMV] were mapped to the tomato (assembly accession GCA_000188115) and tomato mosaic virus (accession FN985165) genome reference sequences. The results are expressed as average values of three repetitions (each composed of four plants) per treatment.

The reads were mapped to the ToMV and tomato genome sequences. The results showed that 34 and 35% represented virus-derived siRNA obtained from dsRNA + ToMV(+) and ToMV libraries, respectively; the remaining sequences corresponded to endogenous siRNA (Fig. 7). In contrast, 97, 96 and 96% of the siRNA molecules in mock, dsRNA, and dsRNA + ToMV(−) libraries, respectively, matched to the tomato sequence (Fig. 7).

**Figure 8:**
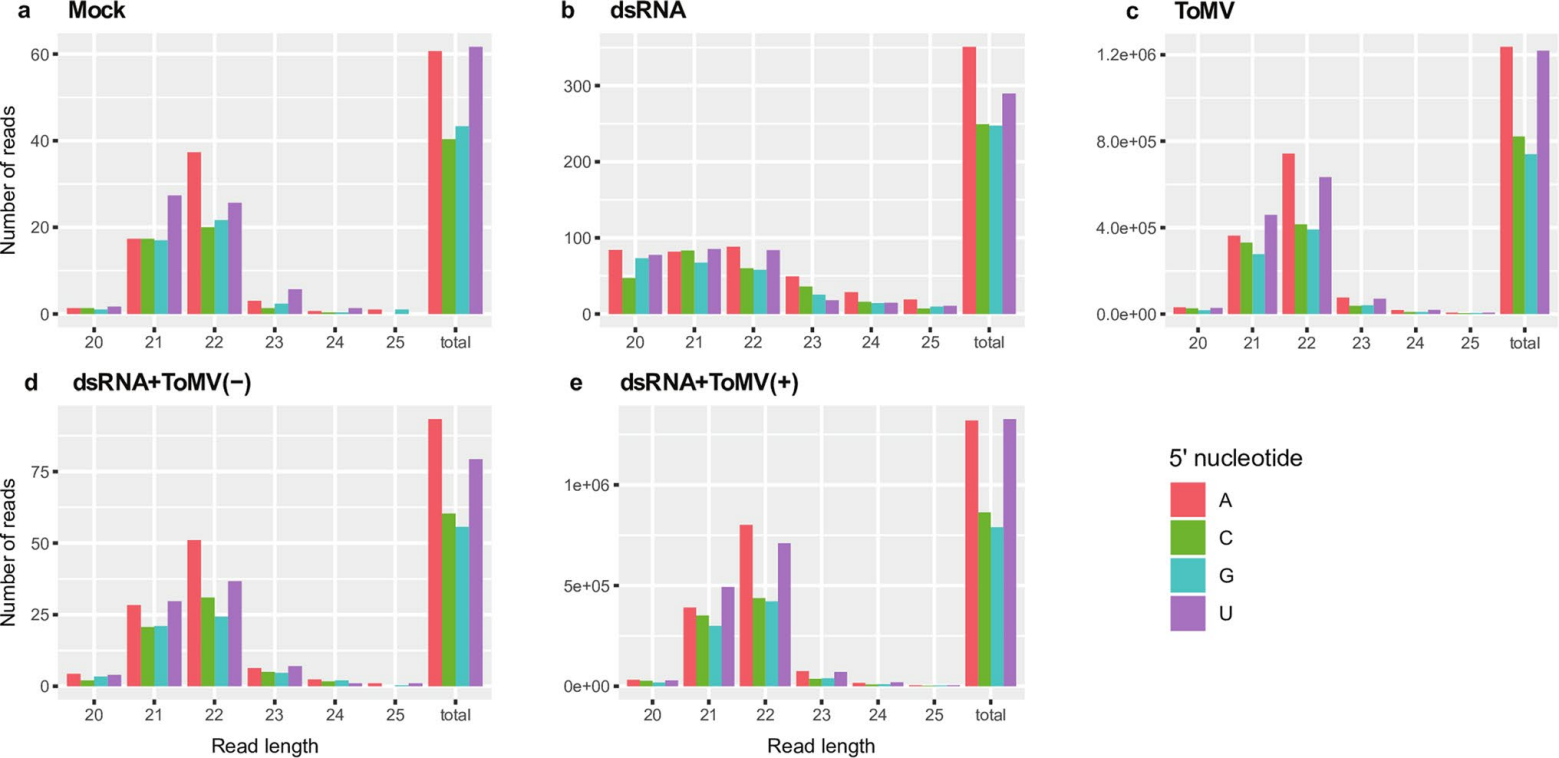
Small interfering RNA (siRNA) analysis in tomato plants, as identified by high throughput sequencing. Counts for each siRNA class according to its size and 5′-nucleotide identity were examined in the five libraries [mock (**a**), dsRNA (**b**), ToMV (**c**), dsRNA + ToMV(−) (**d**), and dsRNA + ToMV(+) (**e**)] using average values from three repetitions (each composed of four plants) per treatment. Columns in the histograms represent the number of reads for each siRNA size class between 20- and 25-nt. Colored bars represent the 5′-nucleotide identity.

The amount of 20- to 25-nt reads in treatments without ToMV [mock, dsRNA, and dsRNA + ToMV(−)] was substantially lower than those containing replicating ToMV [dsRNA + ToMV(+) and ToMV] (Fig. 8). In spite of the variation in the population of siRNA among the libraries, the distribution of the siRNA size class profiles (20- to 25-nt) was basically uniform, differing only in plants treated with dsRNA alone (Fig. 8b). In this case, the amount of 20-, 21-, and 22-nt reads was similar and higher than those of 23-, 24- and 25-nt sequences (Fig. 8b). In the other four libraries, two major siRNA groups were produced containing predominantly 21- and 22-nt siRNA (Fig. 8a,c–e).

The 5′-end nucleotide was analyzed for the most prevalent siRNA classes. The 5′-end U was more abundant in siRNA 21-nt class, while 5′-end A was more abundant in 22-nt class (Fig. 8). In general, the 5′-end G was the least abundant nucleotide (Fig. 8). The 5′-end A and 5′-end U were also more frequent in the sequences from the dsRNA library, although less pronounced than in others (Fig. 8b).

**Figure 9:**
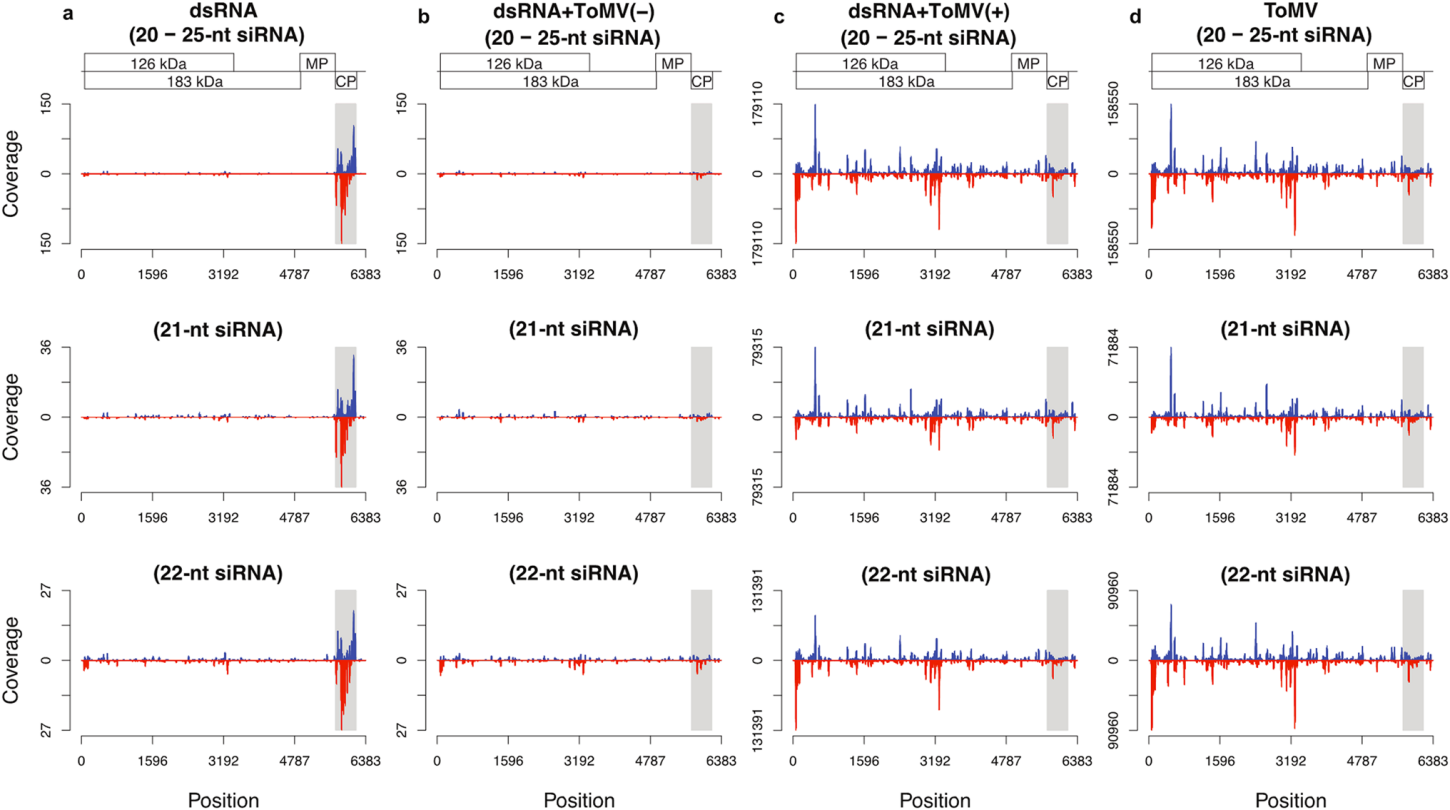
Single-nucleotide resolution maps of small interfering RNA (siRNA) in tomato plants from four libraries: dsRNA (**a**), dsRNA + ToMV(−) (**b**), dsRNA + ToMV(+) (**c**), and ToMV (**d**). Histograms plot the numbers from 20- to 25-nt, 21-nt and 22-nt viral siRNA reads at each ToMV-genome nucleotide position. The results are expressed as average values of three repetitions (each composed of four plants) per treatment. Sense-strand reads are shown above the X axis; antisense-strand reads are shown below the X axis, representing the ToMV genome. Scaled ToMV genome diagram is shown above the histograms, and the corresponding position below the diagram. Y axis represents the coverage in number of reads.

According to the single-nucleotide resolution maps, the major siRNA size classes (21- and 22-nt) covered the entire ToMV genome in both forward and reverse orientations when there was viral infection (Fig. 9c,d). Although the amount of ToMV-specific reads (number of reads from sense- or antisense-sequences for each nucleotide) was slightly higher for dsRNA applied plant samples (Fig. 9c) than in non-applied plants (Fig. 9d), their distribution profile along the genome was essentially the same, suggesting low (or no-) interference of the dsRNA in the virus propagation when the infection was successfully established. In these libraries [dsRNA + ToMV(+) and ToMV], two hotspots of sense and antisense siRNA were observed within the RNA polymerase ORF (Fig. 9c,d). As expected, plants treated only with dsRNA contained the siRNA corresponding to the *cp* gene (Fig. 9a), demonstrating that the topically applied dsRNA molecules were introduced into the plants, processed into siRNAs by endogenous RNAi machinery, and were present in non-treated young leaves. No ToMV-specific siRNA molecules were detected in protected plants, which were exposed to ToMV-dsRNA and ToMV (Fig. 9b), not differing from the mock treatment (data not shown). This indicated a complete absence of virus replication in these plants at the tested time point.

### Failure in triggering protection against ToSRV infection

To examine whether exogenously applied dsRNA molecules have the capacity to confer protection against a phloem-limited DNA virus, ToSRV-dsRNA was applied on the plants following ToSRV inoculation by whiteflies under a high inoculum pressure (~ 30 viruliferous whiteflies per plant). PCR was performed at 14 dpi to confirm infection using universal degenerate primers for begomovirus detection. In three independent trials, none of the 34 inoculated plants were protected against ToSRV (Supplementary Fig. S4), and they showed clear symptoms of interveinal chlorosis. As expected, all plants without dsRNA were infected and those inoculated with aviruliferous whiteflies, and non-inoculated plants were not infected (Supplementary Fig. S4). These results strongly suggest that dsRNA molecules do not induce protection to this particular virus.

## Discussion

RNAi pathways have been exploited as tools for controlling pathogenic viruses in transgenic plants which express self-complementary RNAs (hairpin RNA, hpRNA)^2,27–30^. However, there are some limitations to the use of this strategy, such as the establishment of effective protocols for transformation of important plant crop species. There are also regulatory issues and concerns over the ecological impact of virus-resistant transgenic plants^31^. Since the early 2000s, RNAi-based non-transgenic approaches using exogenously applied dsRNA have been proposed as biotechnological tools for plant viral defense^16^. In the current study, we followed the same strategy and provide data showing that the topical application of dsRNA molecules homologous to *cp* genes from ToMV and PVY genomes induced significant and specific resistance against these viruses in tomato plants. Additionally, we explored the mechanisms behind the siRNA biogenesis generated from these molecules.

ToMV-infected plants usually exhibit strong mosaic and leaf deformation symptoms. Delay in infection or in symptom expression was not observed, either the plants became protected or remained susceptible after dsRNA application. However, ToMV-infected dsRNA-treated plants presented a more vigorous development and milder symptoms than positive controls (Supplementary Fig. S2). This observation indicates that plants primed with dsRNA responded more efficiently against viral infection even in cases where immunity was not achieved. Similar results were reported by Konakalla et al.^23^, who observed an increase in biomass of tobacco plants challenged with tobacco mosaic virus (TMV) and dsRNA from two different TMV genes (*p126* and *cp*).

The results of the ToMV-dsRNA concentration test exhibited a dose-dependent response from 50 to 400 µg/plant (Fig. 2). This suggests that there is a threshold (a minimum required dsRNA amount) for interference in viral infection, once lower concentrations (up to 16 µg/plant) did not induce protection in the plants. In general, the amount of dsRNA employed in different studies involving topical application is highly variable. For instance, Kaldis et al.^24^ used 40 to 60 µg of dsRNA against zucchini yellow mosaic virus (ZYMV) in cucurbits, i.e., up to five times less than in our study. The dose of 200 µg per plant standardized in our trials is in line with 179.2 and 244.8 µg/plant used by Konakalla et al.^23^ against TMV. Most likely the dsRNA concentration that is effective to protect plants against viral infection will vary according to the virus, host, dsRNA purity, content of the solution for delivering the molecules, and finally the modifications done to enhance the application efficiency, stability, and entry into the plant cells.

Topical application of exogenous dsRNA molecules of varying sizes have been previously tested for virus protection in plants^18,23,24,32,33^, including the use of crude nucleic acid extracts from dsRNA/hpRNA-expressing bacterial strains^14,15,19–21^. Additionally, there are different perspectives for use of dsRNA via cell-penetrating peptides^34^ and by high-pressure spraying^35^. Here, we compared some methods for dsRNA application that may be adapted for large-scale use. According to our results, methods involving the addition of abrasive were the most advantageous (Fig. 3), probably because it facilitated access of dsRNA molecules into plant cells, leading to higher resistance induction. The strategy of root immersion in a dsRNA solution for viral control had not been tested previously. Despite its ineffectiveness in inducing plant protection (Fig. 3), the dsRNA absorbed by the roots were detected in leaves (data not shown), which might indicate that dsRNA molecules were not efficiently transported from the xylem to other tissues in the aerial part. It suggests that there is a need for a delivery mechanism into the cell, at least by opening an entrance wound in the cell wall. It is relevant that the protection response was equally efficient by either mechanical inoculation or spraying with the abrasive (Fig. 3); spraying is the most likely method for field application.

We have also observed that co-application of dsRNA and virus is more efficient than pre-treating plants one day prior to virus inoculation (Fig. 5), indicating prompt protection. The protection was transient, even though long-dsRNA molecules were still detectable on treated leaves for at least 10 days (Fig. 6). This indicates that the long-dsRNA only entered into the cells through micro-wounds made during the application process; a system of gradual and continuous introduction of the dsRNA into the cells and/or a long-lasting amplification mechanism of the specific-RNA processing machinery is required for durable and efficient resistance of the plants.

The siRNA systemic movement was observed by HTS; siRNAs were detected in leaves above the application sites. Based on the analysis of single-nucleotide resolution maps, plants treated only with dsRNA presented siRNA molecules concentrated at the ToMV genomic 3′ region, corresponding to the *cp* gene (Fig. 9a). This confirmed that endogenous RNAi machinery successfully recognized and processed the internalized dsRNA. Therefore, the applied dsRNA served as substrate for plant DCL proteins. Conversely, we found no evidence of long-dsRNA systemic transport (Fig. 6), as shown by Tenllado and Díaz-Ruíz^18^. Nevertheless, systemic movement of long-dsRNA was reported by other research groups in non-treated leaves from 9 to 41 dpt^23,24,33^. These results are not compatible with the short durability of protection, in general limited to 2 to 7 dpt^15,19,22^ and contrast with our results showing long dsRNA transport via xylem (uptake by roots).

Distinct from the results obtained for the ToMV-dsRNA and PVY-dsRNA, plants treated with ToSRV-dsRNA were not protected against this begomovirus (Supplementary Fig. S4). We further performed a trial using long-dsRNA cleaved by RNAse III, mimicking processing by DCL that naturally occurs in the RNAi pathway (data not shown). In the two independent experiments, five out of six ToSRV-inoculated plants were infected, indicating a failure to incite a protection response upon the application of either long or small dsRNA molecules against ToSRV. In another study, topical dsRNA application produced plant protection against tomato leaf curl virus (ToLCV, a begomovirus)^33^. In this report, dsRNA of 449 bp and 432 bp including the overlapping part of genes *AC1* and *AC4* and of *AV1* and *AV2*, respectively, were produced; these are genomic fragments that encode coat protein, replication and silencing suppression proteins. In addition, a fusion construct joining *AC1/AC4* with *AV1/AV2* was also tested. These dsRNA solutions were mechanically applied on tomato leaflets immediately after ToLCV agroinfiltration, and conferred 45, 60 and 50% protection, respectively^33^. We speculate that the target genomic region chosen and the method of virus inoculation (here, by whiteflies) may explain the distinct results, although these hypotheses have not been tested. Furthermore, based on the result of the siRNA single-nucleotide resolution maps obtained in the dsRNA library (from ToMV), we observed that the amount of 24-nt fragments (number of reads from sense- or antisense-sequences for each nucleotide) was strikingly lower than those of 21- and 22-nt (Supplementary Fig. S5). Moissiard and Voinnet^36^ suggested that 24-nt siRNA molecules, processed by the DCL3, are involved in the plant protection against a DNA virus, presumably at the transcriptional level. Therefore, the low amount of 24-nt siRNA produced from the topically applied ToSRV-dsRNA, added to a high inoculation pressure, might not have been sufficient to control the ToSRV infection.

The resistance rate of ~ 60% was consistent in ToMV dsRNA-applied plants. In these protected plants, the amount of 20- to 25-nt siRNA molecules was similar to those detected in mock plants (Fig. 8a,d). In contrast, a higher amount of siRNA was observed in plants that received only dsRNA (Fig. 8b). As expected, these molecules were mapped to the CP region of ToMV (Fig. 9a), corresponding to the source dsRNA. Only one out of three biological replicates from the dsRNA library presented 20- to 25-nt siRNA mapped to ToMV-CP in which a larger amount of 20- to 23-nt siRNA was differentially detected (Supplementary Fig. S6b). This variation among the replicates was exclusive to this library (Supplementary Figs. S6 and S7), suggesting that the resistance mechanism was still activated at day 6 in at least one replicate composed of four plants. Mitter et al.^37^ described the detection of small RNA molecules (> 29 bp) in non-treated leaves of *Nicotiana tabacum* plants, 20 days post dsRNA application of cucumber mosaic virus encapsulated in clay nanosheets, while they were not detected when non-encapsulated dsRNA was applied. Here, we could demonstrate the biogenesis of the siRNAs (20–25-nt) in plants after application of long naked dsRNA on a plant surface. We observed that plants respond in a different way, and the success of protection is largely dependent upon the individual ability of producing a minimal amount of siRNA molecules for resistance against the virus infection.

In ToMV-infected plants, the RNAi machinery generated siRNAs from the complete viral genomic sequence with an almost equal abundance of sense and antisense reads along the whole genome (Fig. 9c,d). However, some regions might result in siRNA hotspots^38^. Here, two hotspots of sense and antisense siRNA were identified within the RDR ORF (Fig. 9c,d). In preliminary trials using *C. quinoa* and *N. glutinosa*, dsRNA from *cp* and *mp* genes resulted in a similar protective effect (Fig. 1). We chose the CP coding region for ToMV, and for experiments with PVY. Actually, the *cp* gene has been the preferred to induce viral resistance by dsRNA topical application^14,15,21,23–26^. Pooggin^39^ proposed that the viral genomic regions that do not generate a large quantity of siRNA molecules in virus-infected plants are more promising targets for protection than viral siRNA hotspot regions. If this holds true to our tested systems, the *cp* and *mp* genes were theoretically among the most suitable for inducing strong resistance against ToMV infection, as siRNA hotspots generation in ToMV-infected plants were located in RDR ORF.

It is known that plant-silencing pathways are mediated by multigenic families of DCL and AGO. Our bioinformatics analysis indicated that two major siRNA groups were produced containing predominantly 21- and 22-nt reads in the libraries (Fig. 8); this has been previously observed in watermelons infected by ZYMV^24^. The 5′-nt identities for 21- and 22-nt siRNA showed that 5′U and 5′A were prevalent, whereas the 5′G were less abundant (Fig. 8). Similar results were reported in potatoes infected by PVY and potato virus X^40^.

Recent studies have helped to unravel the identification of DCL, AGO, and RDR families in *S. lycopersicum*^41^ and uncover the mechanisms involved in the endogenous siRNA biogenesis in tomato plants^42,43^. From our data, we infer that exogenous dsRNA molecules were recognized and processed by different DCL proteins, since the siRNA size profile was considerably distinct in the dsRNA library (Fig. 8b). Nevertheless, the distribution of 5′-end siRNA was equivalent in all libraries, being particularly more abundant for 5′U and 5′A (Fig. 8), indicating an association with AGO1 and AGO2, respectively^6,40^. AGO1 and AGO2 are the two major plant antiviral AGO recognized to have a role against RNA viruses in *Arabidopsis*^44^. DCL4 and DCL2, which respectively generate 21- and 22-nt siRNA^4,40^ and play roles in defense against RNA viruses^45,46^, were likely more active in all libraries; in plants treated only with dsRNA (Fig. 8b), which exhibit a slightly different profile, other DCLs could be also involved in the siRNA production.

Further research is needed in order to elucidate whether exogenous dsRNA molecules are actually processed by different plant RNAi-machinery components. Given the variability of RNAi effectiveness for the protection induction against viruses, further tests are extremely important to unravel factors behind the dsRNA-mediated efficiency, contributing to the improvement of control methods and protection durability.

## Methods

### Plant growth conditions and viral inocula

Tomato seedlings (cv. Santa Clara) were transplanted in soil and kept in growth chambers at a constant temperature of 25 °C, 65% RH and 16/8 h light/dark cycle. Individually potted plants were used at the 3- to 4-true leaf stage. Hypersensitive indicator hosts *Chenopodium quinoa* and *Nicotiana glutinosa* plants were used in preliminary assays; they were grown under natural conditions in the greenhouse.

The isolates ToMV-BR01 and PVY-To1 (Embrapa Vegetables collection, Brasília, Brazil) were used in all trials. These viruses were mechanically inoculated on carborundum-dusted leaves by gently rubbing 20 µL of sap from systemically infected tomato leaves. Sap was diluted 1:100,000 and 1:10, in 0.01 M phosphate inoculation buffer at pH 7.0, respectively. The inocula were produced at once, and kept frozen at − 80 °C until use. Batches of the same inocula were used in all trials. Inoculation of the begomovirus ToSRV used the isolate ToSRV-1164^47^ by whitefly (*Bemisia tabaci* Middle East Asia Minor 1) transmission using ~ 30 viruliferous individuals per plant.

### ToMV, PVY and ToSRV target sequences for dsRNA production

Initially, two genomic regions in the coat protein (CP) and the movement protein (MP) of the ToMV were chosen for dsRNA production. In brief, total RNA was extracted from ToMV-infected tomato leaves using Trizol (Thermo Fisher Scientific, Waltham, USA). Viral cDNA was synthesized and the amplicons of 456 bp (CP) and 722 bp (MP) were obtained by PCR amplification reactions employing specific primers (Supplementary Table S1). PCR products from both genes were used for synthesis of dsRNA molecules using MEGAscript RNAi Kit (Thermo Fisher Scientific), following manufacturer's instructions. The concentration and quality of each batch of dsRNA were analyzed by spectrophotometry and 1.2% agarose gel electrophoresis.

The ToMV-dsRNA molecules produced by in vitro transcription were employed in initial tests with *C. quinoa* and *N. glutinosa*, which develop visible local lesions as a hypersensitive response to ToMV infection. Based on the number of lesions, the dsRNA from the CP coding region was selected for all further experiments with ToMV in tomato plants. For ToSRV and PVY, the E-RNAi tool^48^ was used to design the dsRNA from the region comprehending part of both *cp* and *REn* (replication enhancer protein; 496 bp) genes and part of the *cp* gene (481 bp), respectively. Finally, the three selected dsRNA molecules were synthesized by AgroRNA (Seoul, South Korea).

### dsRNA topical application for plant protection against virus infection

ToMV was used as a model virus to standardize some dsRNA application parameters, and the ToMV-dsRNA molecules were employed to test the induction of protection response on tomato plants. The dsRNA solution was applied by gently rubbing on one fully expanded true leaf with abrasive (carborundum, 600-mesh). In preliminary experiments, we applied 0, 1, 4, 8, 16, 50, 100, 200, and 400 µg of dsRNA per plant. After defining the working dose of 200 µg/plant, we tested (i) mechanical application with the abrasive on the second fully developed true leaf, (ii) spray application covering the entire plant with abrasive (carborundum), (iii) spray without abrasive; and (iv) root immersion in dsRNA solution for ~ 3 h prior to transplanting. A brand new individual 5 ml spray bottle atomizer was used for each of the treatments (ii) and (iii). In a similar manner, PVY homologous dsRNA (200 µg/plant) was mechanically applied to test resistance capacity against PVY infection and the specificity of the protection.

After 24 h of dsRNA application, the plants were challenged by mechanical inoculation of the viruses. Plants inoculated with the virus without the dsRNA application, and mock-inoculated plants (phosphate buffer) were used as positive and negative controls, respectively. Each test consisted of 10 to 12 plants per treatment, replicated at least three times. The infection was confirmed for ToMV and PVY at 7- and 10-dpi, respectively, by indirect-ELISA and symptom evaluation. The ELISA test was carried out on a dot-blot format. Briefly, the plant crude sap was applied onto a nitrocellulose membrane, and treated with anti-ToMV or anti-PVY IgG, followed by anti-rabbit alkaline phosphatase conjugated goat antibody (Sigma-Aldrich, Saint Louis, USA), and detected using a chromogenic substrate. A positive detection was observed as clear purple precipitate, while the negative samples remained greenish. The infection rate was determined as the number of ELISA-positive samples out of the total number of plants.

In the ToSRV trials, ToSRV-dsRNA molecules (200 µg/plant) were mechanically applied on all leaves 24 h prior to ToSRV challenge inoculation using ~ 30 viruliferous whiteflies in three independent assays. Whiteflies acquired the virus by feeding on ToSRV-infected tomatoes for 48 h. Then, they were transferred to cages containing healthy plants coated with dsRNA and confined for 72 h for virus transmission. Plants exposed only to viruliferous whiteflies (i.e., without dsRNA), aviruliferous whiteflies, and dsRNA only were used as controls. PCR was performed 14 dpi to confirm infection using universal degenerate primers for begomovirus detection, pAL1v1978 and pAR1c496^49^. All treatments were compared using ANOVA and Tukey’s post-hoc test using Sisvar 5.7.

### Durability of the RNAi-mediated viral protection after dsRNA topical application

To evaluate the durability of the protective effect against ToMV following ToMV-dsRNA mechanical application, ToMV was inoculated in the two youngest fully expanded true leaves at daily time points from 0 to 7 dpt. Time zero means that the dsRNA was applied simultaneously with the virus inoculum (co-application). All plants were prepared at once, hence the inoculation dates and sample collection dates varied according to the treatment. The positive control consisted of plants inoculated with ToMV without dsRNA, used to evaluate the inoculum quality. The infection was tested at 7 dpi by indirect-ELISA. Tests were performed in two independent trials with 10 to 12 plants.

### Testing the systemic transport of long-dsRNA

To analyze the dsRNA translocation *in planta*, ToMV-dsRNA molecules were mechanically applied on one leaf, which was isolated from the other leaves by a barrier made of aluminum foil. DEPC-treated water was applied in negative control plants. Young non-treated and treated leaves were removed from the plants 1 and 6 hpt, and daily from 1 to 10 dpt. Each sample was collected from a new plant to avoid inciting any damage to the plants, and to ensure the collection of the necessary amount of tissue for each analysis. The trial was performed with three replicates for each evaluated time point. Total RNA extraction was performed using Trizol (Thermo Fisher Scientific); cDNA was synthetized employing random hexamer primers. ToMV-dsRNA molecules were detected from treated and non-treated leaves by PCR using specific primers (Supplementary Table S1). The primers 9F and 13R for the chloroplast gene transcript^50^ were used as the endogenous control.

### ToMV-specific siRNA analysis by high throughput sequencing (HTS)

The siRNA population originating from ToMV was analyzed in plants that were protected from ToMV infection by dsRNA application and in non-protected plants. ToMV-dsRNA was mechanically applied on one leaf, followed by ToMV inoculation in the same leaf 24 hpt. Sampling was performed six days after dsRNA application in plants, but before symptom expression. For siRNA HTS, the tests were carried out with five treatments in three independent trials: (i) ToMV-dsRNA application + ToMV inoculation (resulting in ToMV-infected plants), (ii) ToMV-dsRNA application + ToMV inoculation (non-infected plants), (iii) ToMV-dsRNA + inoculation buffer, (iv) DEPC-water + ToMV, and (v) non-treated plants. The ToMV infection was confirmed by indirect-ELISA. From each sample, a mixture of total RNA and siRNA was obtained for sequencing. First, total RNA was extracted from non-treated leaves of four biological replicas using *mir*Vana PARIS kit (Thermo Fisher Scientific), according to manufacturer’s instructions. Separately, extractions targeting siRNA (< 200 nt) were also carried out with the *mir*Vana PARIS kit. After combining both extracts (total RNA + siRNA), extractions were further purified using RNA clean and concentrator-5 kit (Zymo Research, Irvine, USA); siRNA sequencing was performed on an Illumina HiSeq 2500 platform at Macrogen Inc. (Seoul, South Korea) employing TruSeq small RNA library prep kit for cDNA library preparation, totaling 15 libraries (five treatments and three independent replicates).

Adapter sequences were removed from the reads using BBDuk v38.22 (http://sourceforge.net/projects/bbmap/; command line arguments: ktrim = r k = 21 ref = adapters.fa), and the reads were simultaneously mapped to the tomato (assembly accession GCA_000188115) and ToMV (accession number FN985165) genomes employing BWA aln v0.7.17 tool^51^ (command line arguments: -n1 -o0 -e0 -k1). Sequencing depth along the ToMV genome and counts for each siRNA class according to its size and 5′-nucleotide identity were obtained using SAMTools v1.9^52^ and in-house scripts. Replicates were analyzed independently and their average values were used per treatment for figure generation.

### Equipment and settings

Photographs of plants were taken using Nikon Coolpix P510, and no processing was made except for cropping to adjust to the composite picture in Fig. 1. Graphs were produced using the software Excel (Microsoft Office) version 10 (Figs. 2–5 and 7). The agarose gels were photographed by using Loccus L-PIX imaging system (Fig. 6). Plots were produced with R v.3.6.2 using in-house scripts (Figs. 8 and 9).

## Supplementary Information

Supplementary Information.

## Data Availability

The high-throughput sequencing data generated during the current study are available in the NCBI repository, http://www.ncbi.nlm.nih.gov/bioproject/623982.
