# Supplementary material for: siRNA biogenesis and advances in topically applied dsRNA for controlling virus infections in tomato plants
Source: Sci Rep. 2020 Dec 17;10:22277. doi: 10.1038/s41598-020-79360-5 (PMC7746768; doi:10.1038/s41598-020-79360-5)
Supplement: Supplementary file 1 — Supplementary Information. [file 41598_2020_79360_MOESM1_ESM.docx]

**siRNA biogenesis and advances in topically applied dsRNA for controlling virus infections in tomato plants**

Camila M. Rêgo-Machado^1,2^, Erich Y. T. Nakasu^2*^, João M. F. Silva^3^, Natália Lucinda^2†^, Tatsuya Nagata^3^, Alice K. Inoue-Nagata^1,2*^

^1^Department of Plant Pathology, University of Brasília, Federal District, Brazil.

^2^Laboratory of Virology and Molecular Biology, Embrapa Vegetables, Federal District, Brazil.

^3^Department of Molecular Biology, University of Brasília, Federal District, Brazil.

^†^Present address: Department of Plant Pathology, University of Florida, Florida, USA.

^*^Corresponding author e-mail: [erich.nakasu@embrapa.br](mailto:erich.nakasu@embrapa.br); alice.nagata@embrapa.br

**Supplementary Table S1.** Primers used for production and detection of dsRNA homologous to tomato mosaic virus (ToMV) sequence.


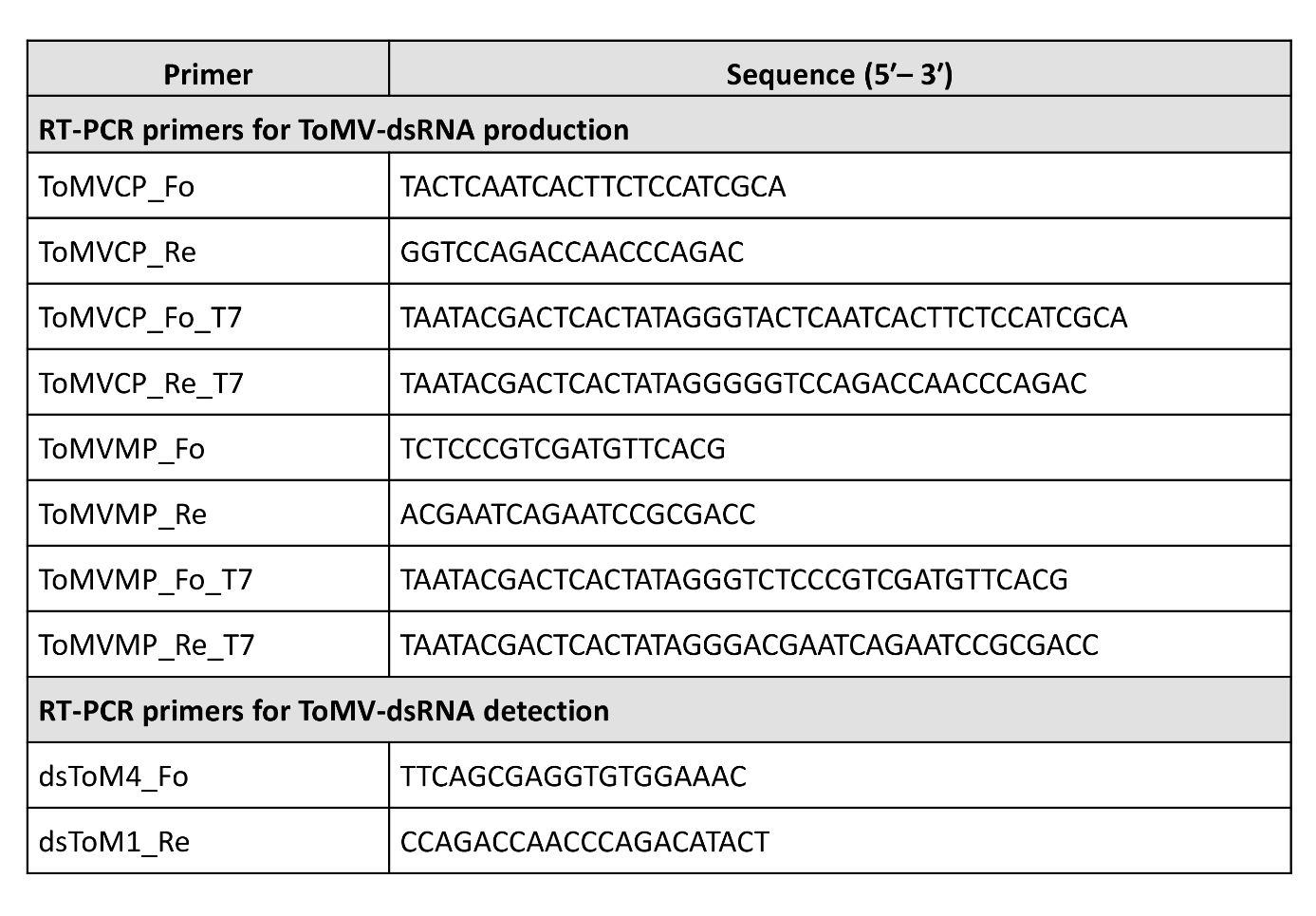


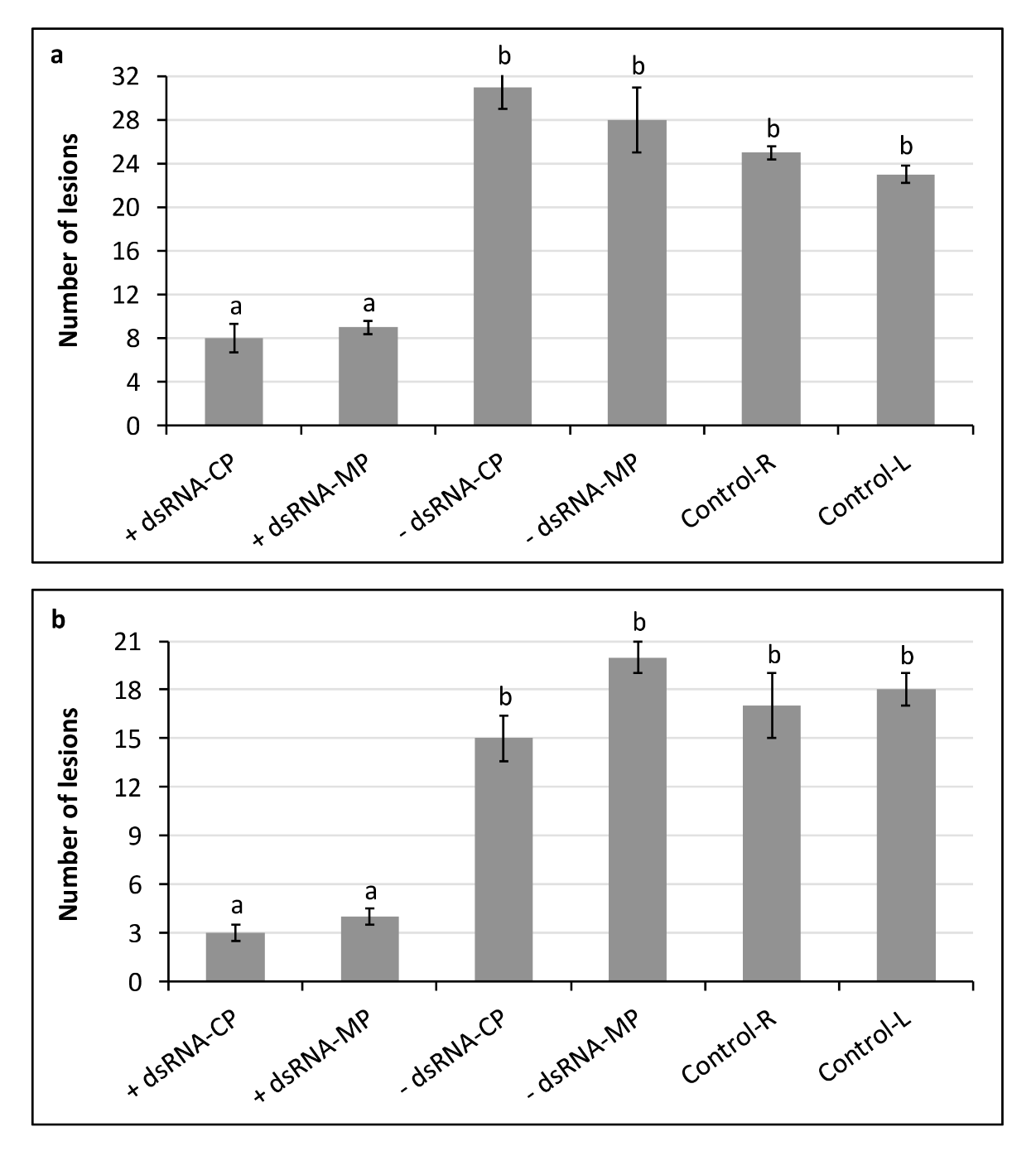


**Supplementary Figure S1.** Effects of dsRNA homologous to tomato mosaic virus (ToMV) sequence 4 days after ToMV mechanical inoculation. The graphs show the number of local lesions caused by ToMV in *Nicotiana glutinosa* (a) and *Chenopodium quinoa* (b). dsRNA of ToMV-*cp* gene (+ dsRNA-CP) and ToMV-*mp* gene (+ dsRNA-MP) were mechanically applied on the left halves simultaneously with ToMV; the right halves were treated with DEPC-water (- dsRNA-CP and - dsRNA-MP) and ToMV. As a control, only ToMV was inoculated on both right (control-R) and left (control-L) halves of the leaves. The results are expressed as average values of 3 plants in 2 independent trials. Bars represent the respective standard errors. Letters above error bars indicate significantly different results based on Tukey test (p < 0.05). Graph prepared using Excel (Microsof Office) version 10.


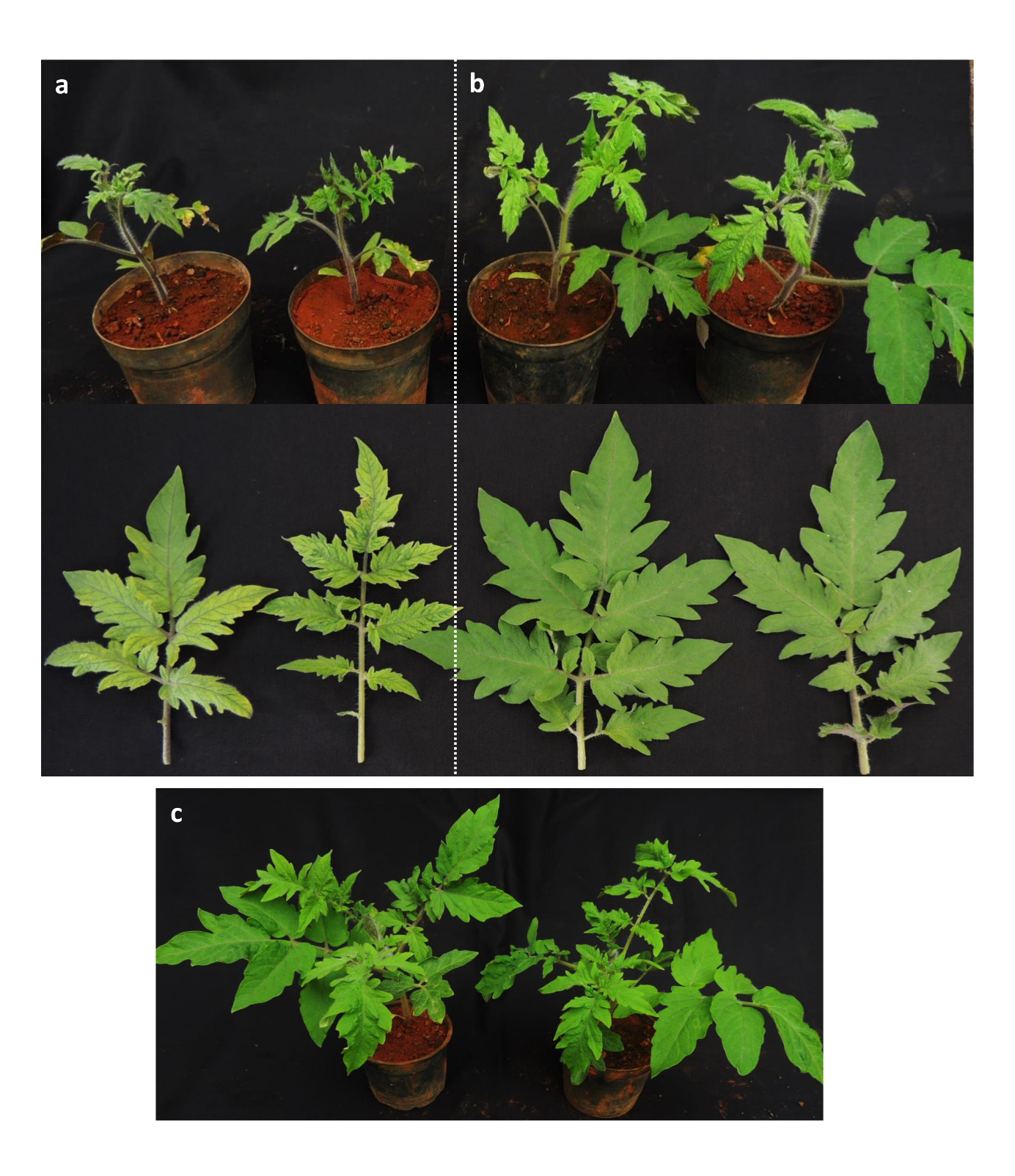


**Supplementary Figure S2.** Effects of dsRNA homologous to tomato mosaic virus (ToMV) sequence on the growth of tomato plants at 15 dpi. dsRNA molecules were mechanically applied on the plants at a dose of 200 µg per plant, and 24 hours later challenged with ToMV. (a) dsRNA-untreated ToMV-infected plants (positive control). (b) dsRNA-treated ToMV-infected plants. (c) dsRNA-treated plants. dsRNA-treated ToMV-infected plants showed a more vigorous development and milder disease symptoms compared to the positive control plants. Pictures (a) and (b) are in the same scale. Photographs were taken using Nikon Coolpix P510, and no processing was made except for cropping to adjust to the composite figure.


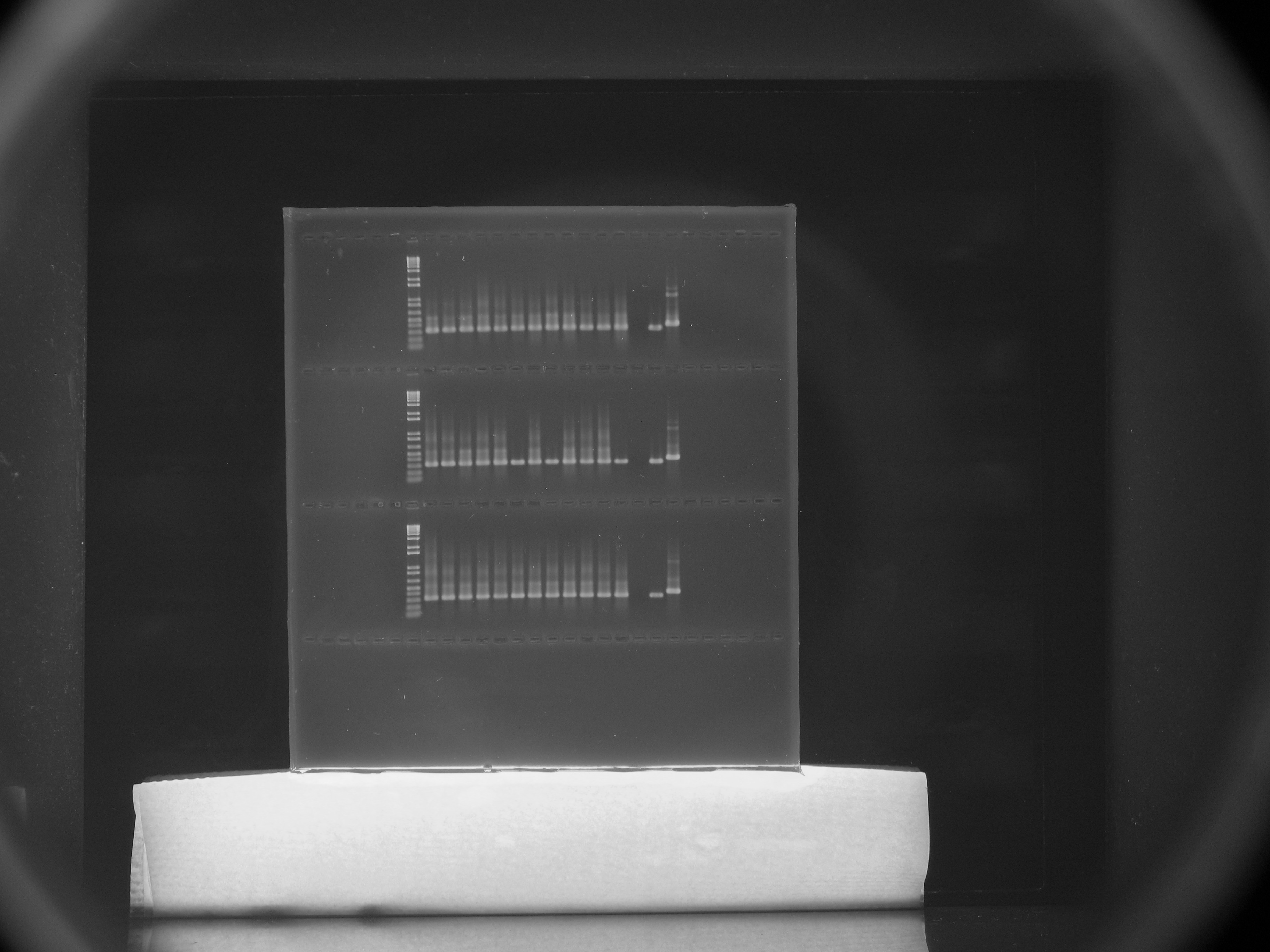

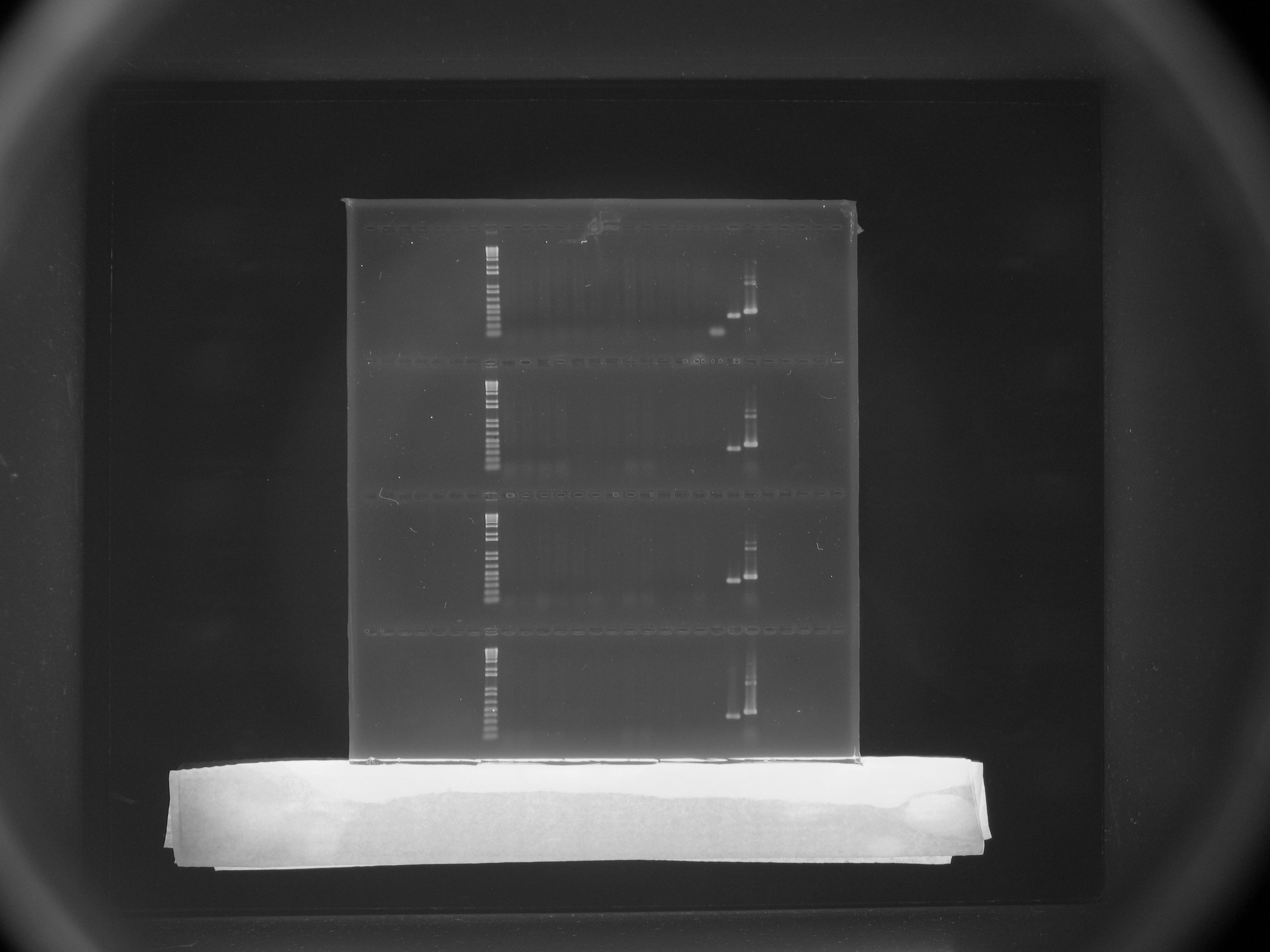


**a**

**b**

**M**

**1H**

**6H**

**1D**

**2D**

**3D**

**4D**

**5D**

**6D**

**7D**

**8D**

**9D**

**10D**

**-C**

**+C**

**EC**

**M**

**1H**

**6H**

**1D**

**2D**

**3D**

**4D**

**5D**

**6D**

**7D**

**8D**

**9D**

**10D**

**-C**

**+C**

**EC**

**Supplementary Figure S3.** Expanded data supplementary figure containing two gels used to compose Fig. 6. Ethidium bromide stained 1.2% agarose gel with RT-PCR products from total RNA extracted from dsRNA treated (a) and non-treated (b) tomato leaves for detection of the long dsRNA at different time points after application [1 and 6 hours (H), and daily from 1 to 10 days (D)]. Three biological repetitions were tested from treated leaves and depicted in panel (a), plant 1, plant 2, and plant 3, from top to bottom. Three biological repetitions were also tested from non-treated leaves, and mock (without dsRNA) samples in panel (b), plant 1, plant 2, plant 3, and mock, from top to bottom. M: molecular weight DNA ladder (1 Kb plus DNA ladder, Thermo Fisher Scientific). –C: RT-PCR negative control (DEPC-water). +C: RT-PCR positive control (ToMV-dsRNA). EC: endogenous control (spliced chloroplast transcript). Agarose gels photographed using Loccus L-PIX imaging system. A few non-specific amplicons were observed but this did not interfere with the analyses.


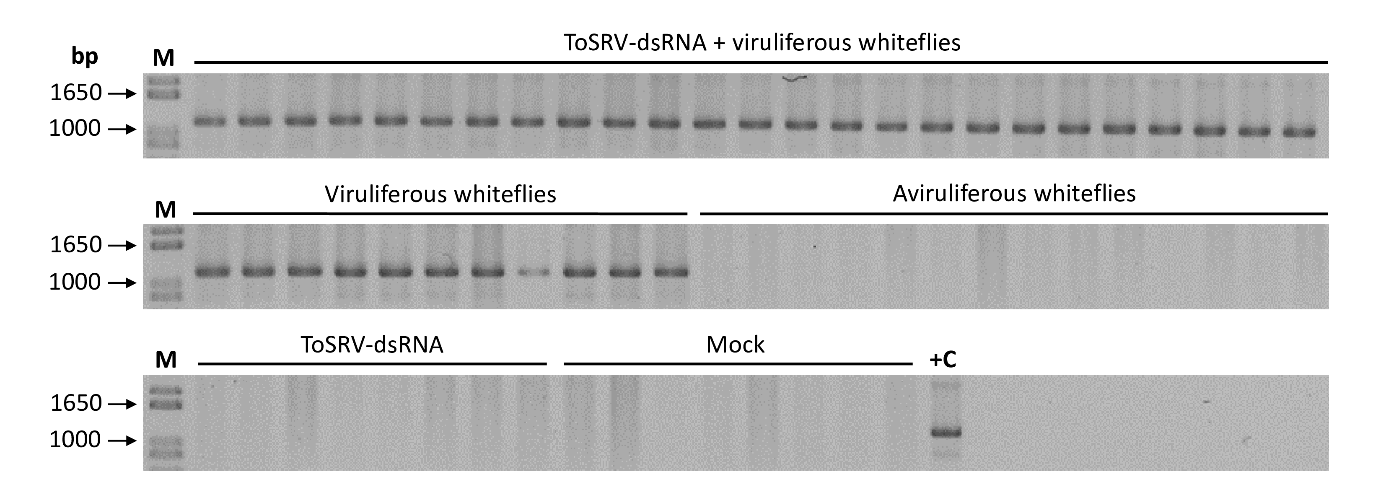

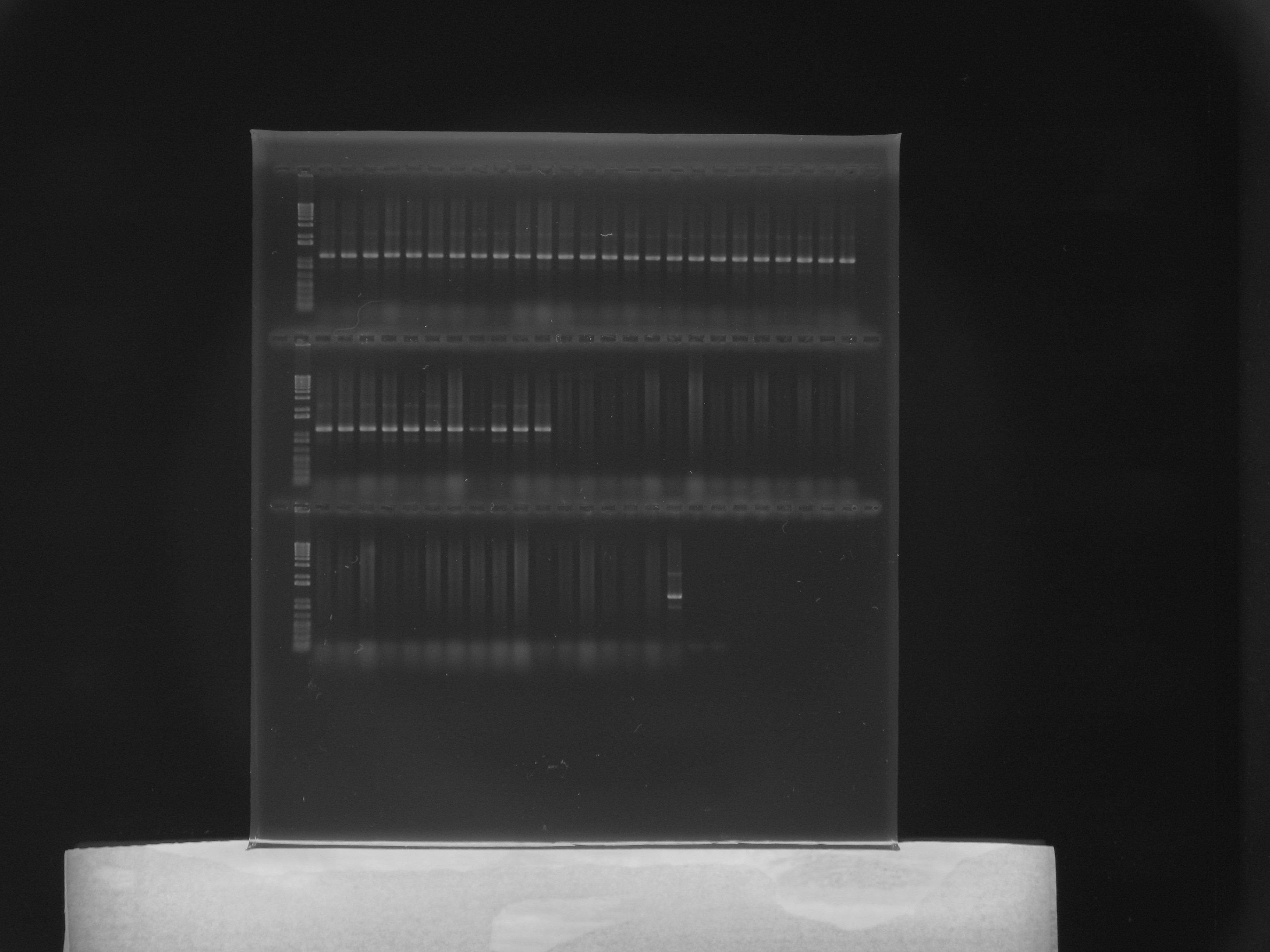


**a**

**b**

**Supplementary Figure S4.** Detection of tomato severe rugose virus (ToSRV) by PCR using universal primers for begomovírus in tomato plants. This figure shows a representative electrophoresis gel from samples collected at 14 dpi. ToSRV-dsRNA molecules were mechanically applied on plants 24 hours prior to ToSRV inoculation by ~30 viruliferous *Bemisia tabaci* MEAM1. (a) First panel: all plants became infected after ToSRV inoculation on ToSRV-dsRNA treated leaves. Second panel: plants inoculated with viruliferous whiteflies, and aviruliferous whiteflies. Third panel: plants treated only with dsRNA, and mock control. M: 1 Kb Plus DNA ladder (Thermo Fisher Scientific). +C: PCR positive control (ToSRV-infected tomato leaves). (b) Original electrophoresis agarose gel picture used to produce the composite figure in (a). Agarose gel photographed using Loccus L-PIX imaging system.


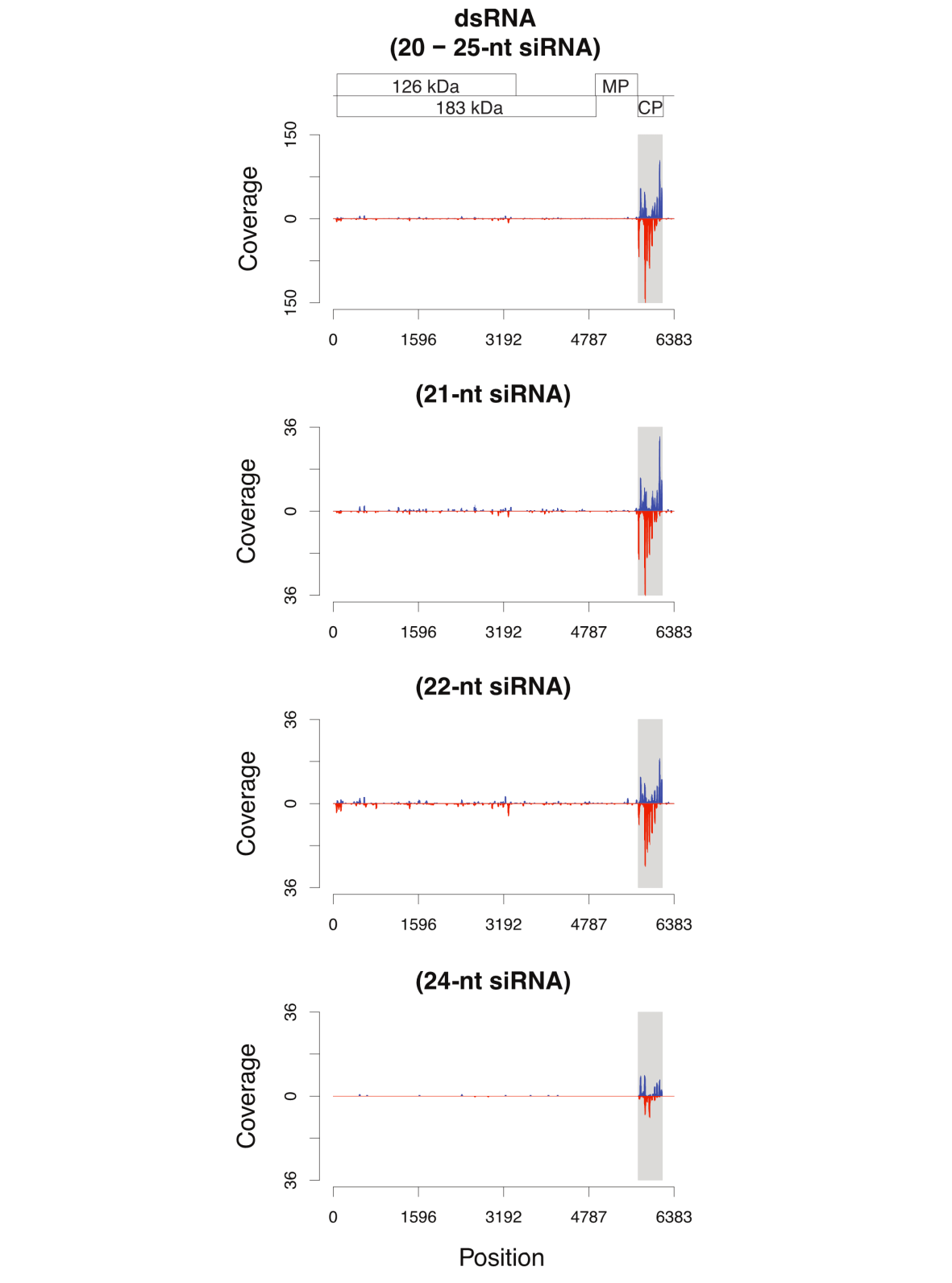


**Supplementary Figure S5.** Single-nucleotide resolution maps of small interfering RNA (siRNA) in tomato plants from the dsRNA library. Histograms plot the numbers from 20- to 25-nt, 21-nt, 22-nt and 24-nt viral siRNA reads at each ToMV-genome nucleotide position. The results are expressed as average values of three repetitions (each composed by four plants) per treatment. Sense-strand reads are shown above the X axis; antisense-strand reads are shown below the X axis, representing the ToMV genome. Scaled ToMV genome diagram is shown above the histograms, and the corresponding position below the diagram. Y axis represents the coverage in number of reads. Plots were produced with R v.3.6.2 using in-house scripts.


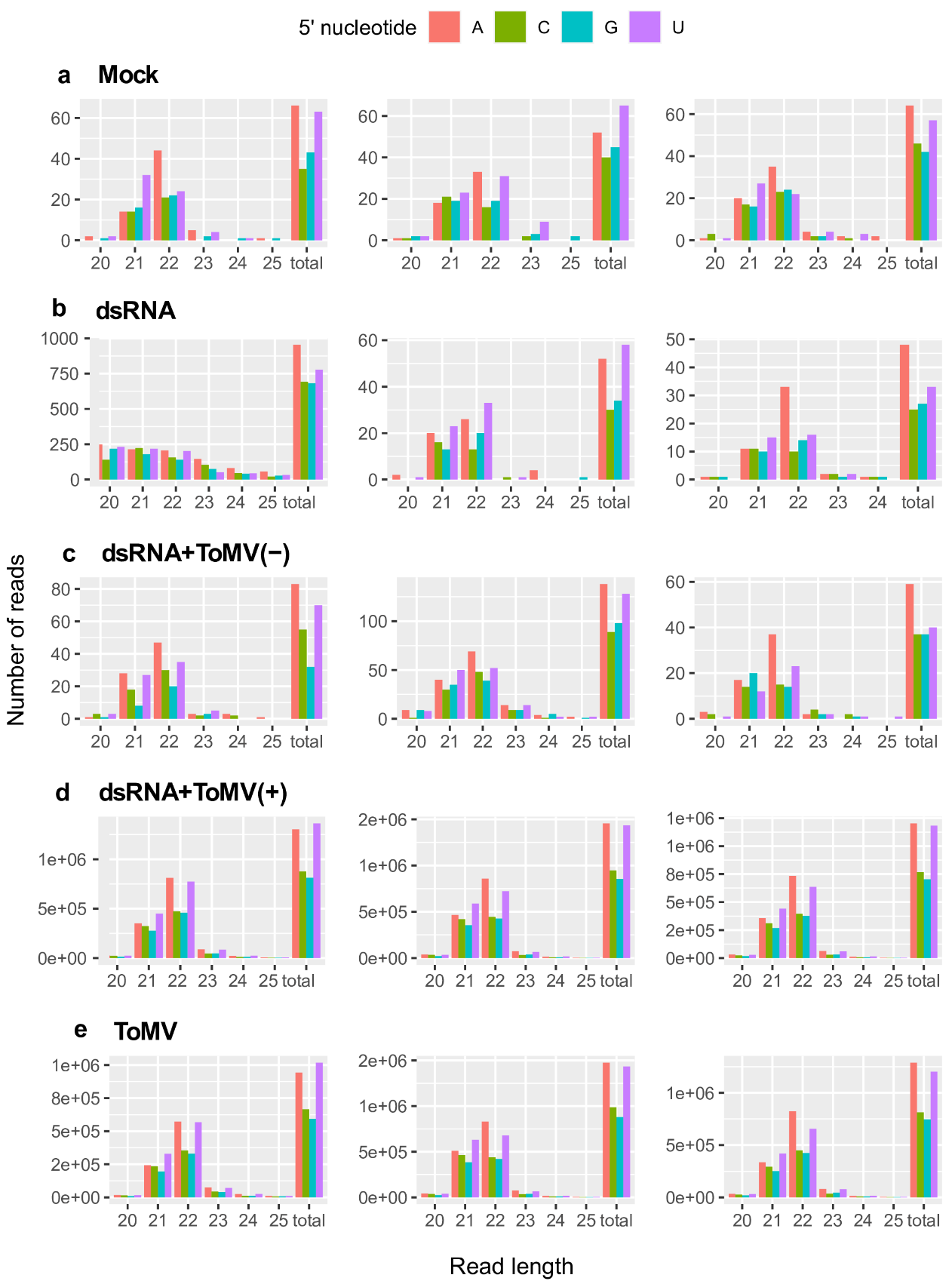


**Supplementary Figure S6.** Small interfering RNA (siRNA) analysis in the three repetitions of tomato plants, as identified by high throughput sequencing. Counts for each siRNA class according to its size and 5´-nucleotide identity were examined separately in the three repetitions (each composed by four plants) from the five libraries: mock (a), dsRNA (b), dsRNA+ToMV(-) (c), dsRNA+ToMV(+) (d), and ToMV (e). Columns in the histograms represent the number of reads for each siRNA size class between 20- and 25-nt. Colored bars represent the 5´-nucleotide identity. Plots were produced with R v.3.6.2 using in-house scripts.


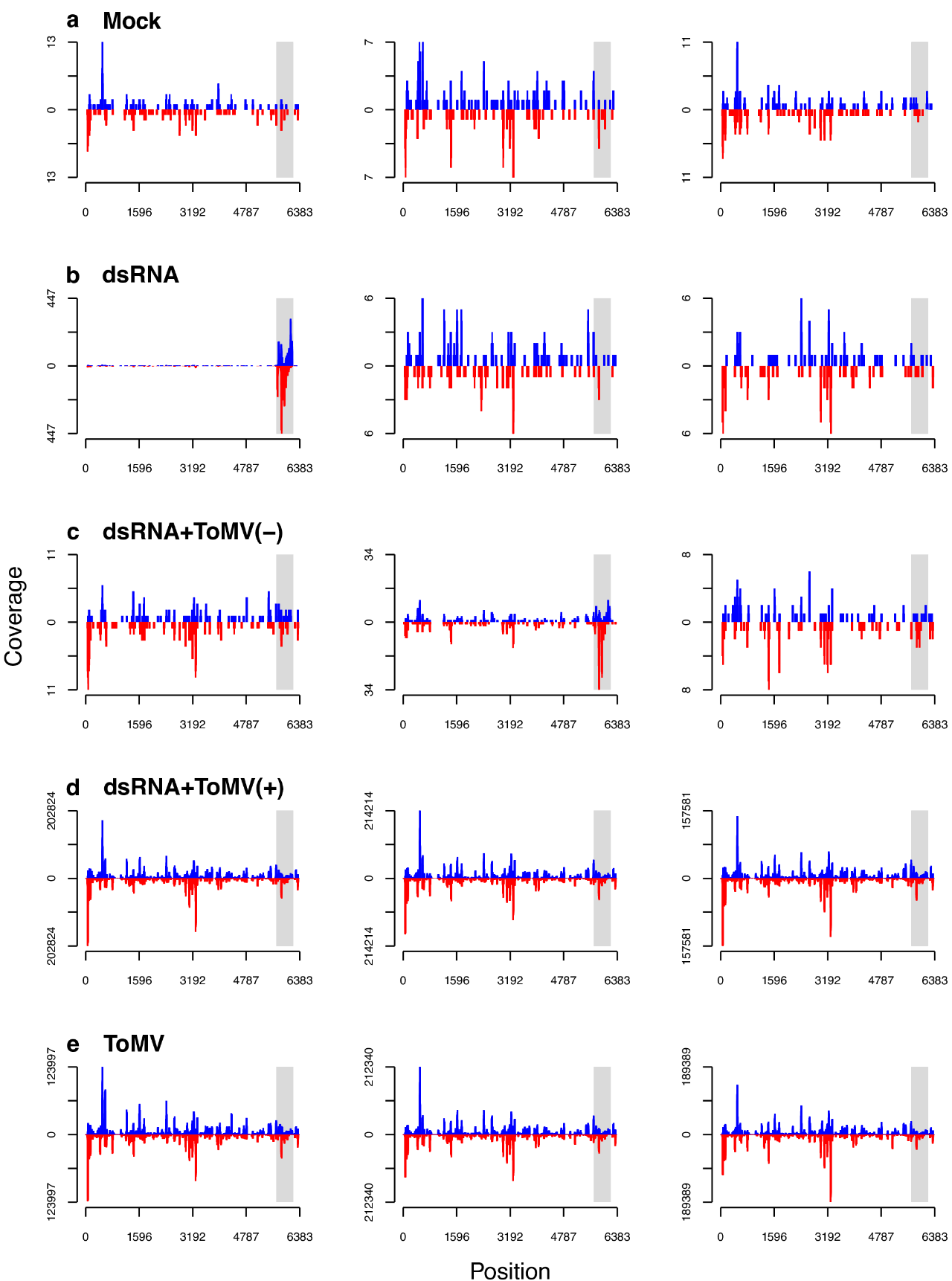


**Supplementary Figure S7.** Single-nucleotide resolution maps of small interfering RNA (siRNA) in the three repetitions of tomato plants from the five libraries: mock (a), dsRNA (b), dsRNA+ToMV(-) (c), dsRNA+ToMV(+) (d), and ToMV (e). Histograms plot the numbers from 20- to 25-nt viral siRNA reads at each ToMV-genome nucleotide position. The results were examined separately in the three repetitions (each composed by four plants) per treatment. Sense-strand reads are shown above the X axis; antisense-strand reads are shown below the X axis, representing the ToMV genome. Y axis represents the coverage in number of reads. Plots were produced with R v.3.6.2 using in-house scripts.
